# Supplementary material for: Deleting the mitochondrial respiration negative regulator MCJ enhances the efficacy of CD8+ T cell adoptive therapies in pre-clinical studies
Source: Nat Commun. 2024 May 24;15:4444. doi: 10.1038/s41467-024-48653-y (PMC11126743; doi:10.1038/s41467-024-48653-y)
Supplement: Supplementary file 1 — Supplementary Information [file 41467_2024_48653_MOESM1_ESM.pdf]

Deleting the mitochondrial respiration negative regulator MCJ enhances the efficacy of CD8$^+$ T cell adoptive therapies in pre-clinical studies

Meng-Han Wu, Felipe Valenca-Pereira, Francesca Cendali, Emily L. Giddings, Catherine Pham-Danis, Michael C. Yarnell, Amanda J. Novak, Tonya M. Brunetti, Scott B. Thompson, Jorge Henao-Mejia, Richard A. Flavell, Angelo D'Alessandro, M. Eric Kohler, Mercedes Rincon.

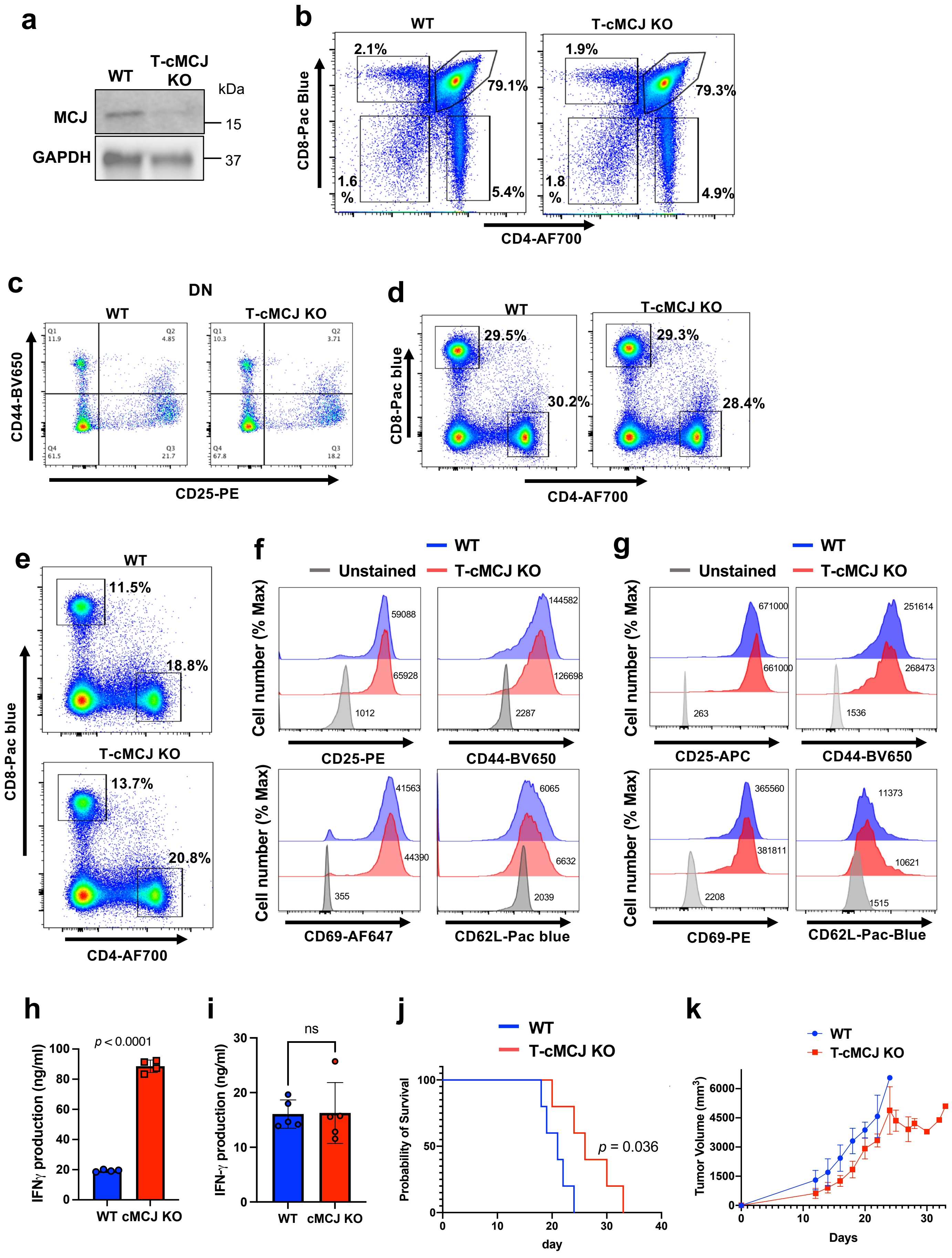

a
WT
T-cMCJ KO
kDa
MCJ
15
GAPDH
37
b
WT
T-cMCJ KO
2.1%
79.1%
1.6 %
5.4%
1.9%
79.3%
1.8 %
4.9%
CD8-Pac Blue
CD4-AF700
c
DN
WT
T-cMCJ KO
CD44-BV650
CD25-PE
d
WT
T-cMCJ KO
29.5%
30.2%
29.3%
28.4%
CD8-Pac blue
CD4-AF700
e
WT
11.5%
18.8%
T-cMCJ KO
13.7%
20.8%
CD8-Pac blue
CD4-AF700
f
WT
Unstained
T-cMCJ KO
Cell number (% Max)
59088
65928
1012
144582
126698
2287
CD25-PE
CD44-BV650
41563
44390
355
6065
6632
2039
CD69-AF647
CD62L-Pac blue
g
WT
Unstained
T-cMCJ KO
671000
661000
263
251614
268473
1536
CD25-APC
CD44-BV650
365560
381811
2208
11373
10621
1515
CD69-PE
CD62L-Pac-Blue
h
p < 0.0001
IFNγ production (ng/ml)
WT cMCJ KO
i
ns
IFN-γ production (ng/ml)
WT cMCJ KO
j
WT
T-cMCJ KO
p = 0.036
Probability of Survival
day
k
WT
T-cMCJ KO
Tumor Volume (mm³)
Days

Supplementary Fig.1: MCJ deficiency in T cells does not affect T cell development but enhances CD8 cell effector function and anti-tumor immunity. (**a**) CD8 cells were isolated from the spleen and lymph nodes of $MCJ^{fl/fl}/CD4\text{-}Cre^{-}$ (WT) and $MCJ^{fl/fl}/CD4\text{-}Cre^{+}$ (T-cMCJ KO) mice. MCJ expression in CD8 cells was examined by western blot analysis. (**b-c**) Thymocytes were isolated from WT and T-cMCJ KO mice and examined by flow cytometry for CD4 and CD8 in the total population (**b**), or for CD25 and CD44 expression within $CD4^{-}CD8^{-}$ (DN) subpopulation (**c**). (**d-e**) CD4 and CD8 cell populations were examined in lymph nodes (**d**) and spleen (**e**) from WT and T-cMCJ KO mice by flow cytometry. (**f**) CD8 cells from spleen and lymph nodes of WT and T-cMCJ KO mice were activated with anti-CD3/anti-CD28 Abs for 48h and expression of activation markers (CD69, CD44, CD25, CD62L) were examined by flow cytometry. MFI is shown. (**g**) CD4 cells from WT and T-cMCJ KO mice were activated as in (f) and expression of activation markers (CD69, CD44, CD25, CD62L) was examined by flow cytometry. MFI is shown (**h**) CD8 cells from spleen and lymph nodes of WT and T-cMCJ KO mice were activated as in (f) for 48h, washed, and incubated in medium alone for 4h. IFNγ levels in the supernatants was determined by ELISA (n=4 biologically independent samples). (**i**) CD4 cells from the spleen and lymph nodes of WT and T-cMCJ KO mice were activated as in (f) for 48h. The level of IFNγ in the culture was determined by ELISA (n=5 biologically independent samples). (**j-k**) B16-OVA melanoma cells (4×10^5/mouse) were s.c. injected on the flank to WT or T-cMCJ KO mice (n=5 mice). Survival of the mice was followed over time (**j**). The tumor size of the mice was measured over time (**k**). *p* was determined by two-sided unpaired t-test (**h, i**) and Mantel-Cox test (**j**). Mean±SD is shown for (**h, I,k**).

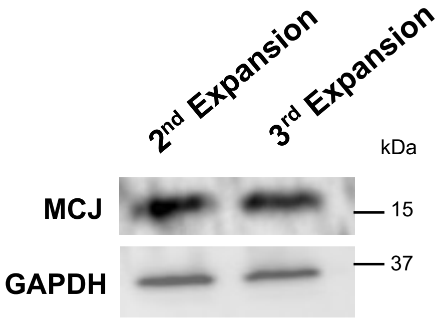

Supplementary Fig.2: MCJ expression is maintained in CD8 cells during IL-2 expansions. WT CD8 cells were isolated from the spleen and lymph nodes, activated with anti-CD3/anti-CD28 beads for 3 days, and expanded with IL-2 for 2 or 3 expansions. MCJ expression in IL-2-expanded CD8 cells was examined by western blot analysis.

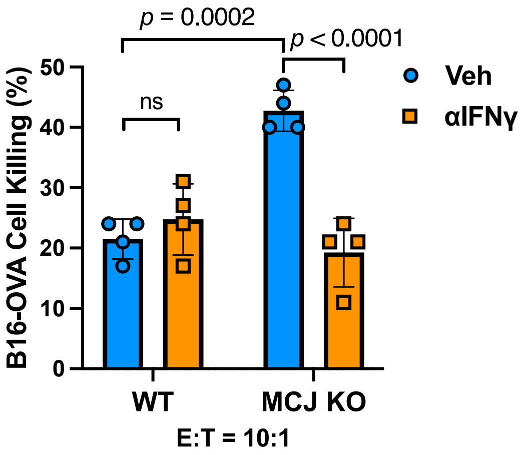

Supplementary Fig. 3: IFNγ blockage abolishes the superior killing activity of MCJ-KO OT-I CD8 cells. WT and MCJ KO OT-I CD8 cells were activated as described in Fig. 1, expanded with IL-2, and after 3 expansions, they were co-cultured with B16-OVA cells at an E:T = 10, in the presence or absence of a blocking anti-IFNγ Ab (5μg/ml) for killing assay (n=4 biologically independent cells). Lived B16-OVA cells were counted after 24 h. Mean±SD is shown. *p* was determined by 2-way ANOVA.

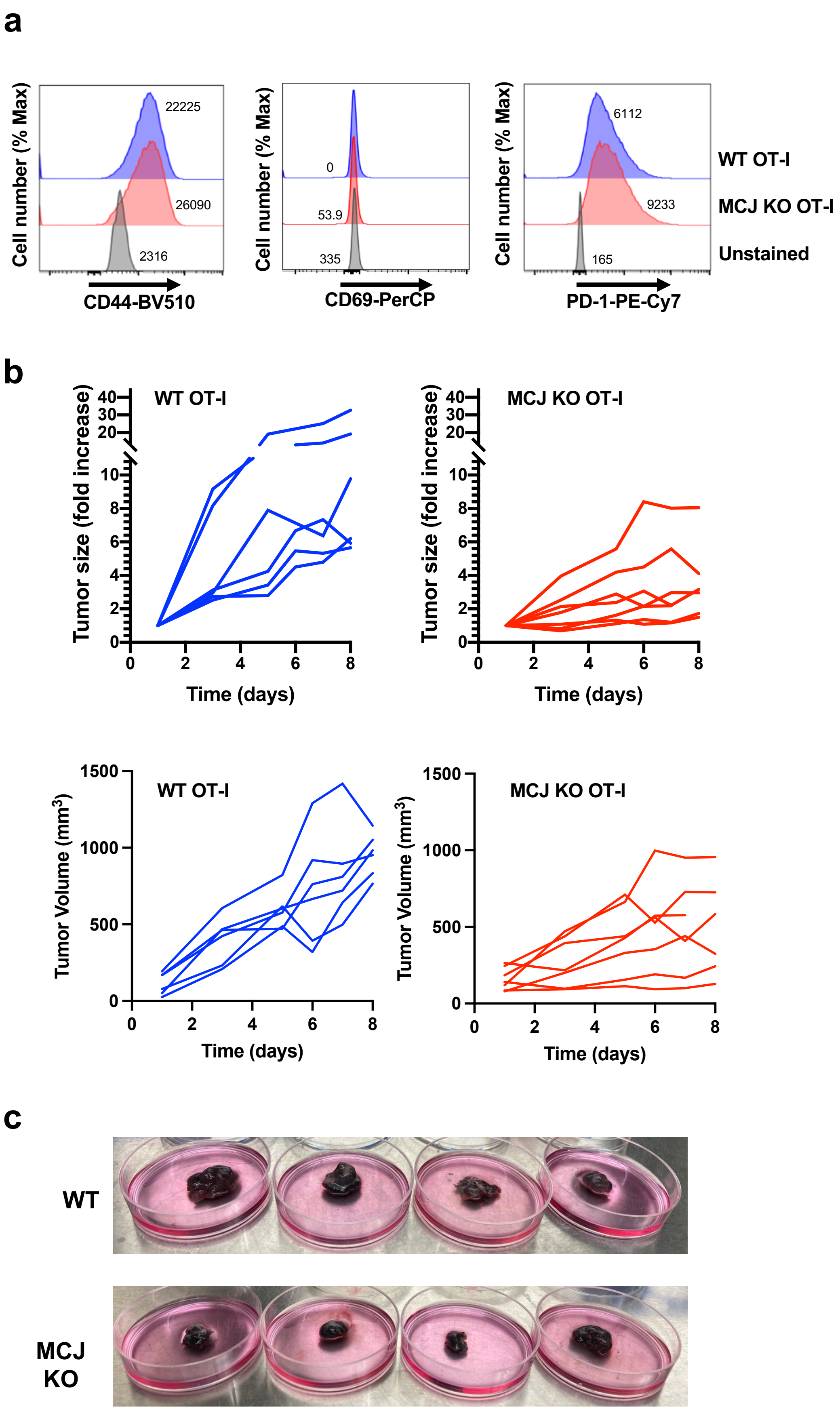

a
Cell number (% Max)
22225
26090
2316
CD44-BV510
0
53.9
335
CD69-PerCP
6112
9233
165
PD-1-PE-Cy7
WT OT-I
MCJ KO OT-I
Unstained
b
WT OT-I
MCJ KO OT-I
Tumor size (fold increase)
Time (days)
Tumor Volume (mm^3)
Time (days)
c
WT
MCJ KO

Supplementary Fig. 4: Superior anti-tumor response of MCJ KO TCR-specific CD8 cells against melanoma *in vivo.* (a-c) B16-OVA tumor cells (4 x 10^5/mouse) were s.c. injected on the flank to WT mice. WT and MCJ KO OT-I CD8 cells were activated with anti-CD3/anti-CD28 Abs, expanded with IL-2 (40 IU/ml) for two expansions, and (5 x 10^5/mouse) i.v. administered to the B16-OVA tumor-bearing mice 10 days post-implantation when the tumors were palpable. (**a**) Activation cell surface markers (CD44, CD69, and PD1) of WT (blue) and MCJ KO (red) OT-I CD8 cells prior to being adoptively transferred to the tumor-bearing mice, examined by flow cytometry. Histograms for unstained MCJ KO OT-I cells are also shown (gray). MFI is shown. (**b**) Tumor volume change (fold increase or tumor volume) in individual mice that had received either WT OT-I cells or MCJ KO OT-I cells (n=6 mice). (**c**) B16-OVA tumors isolated from tumor-bearing mice after 8 days of post-OT-I cells injection.

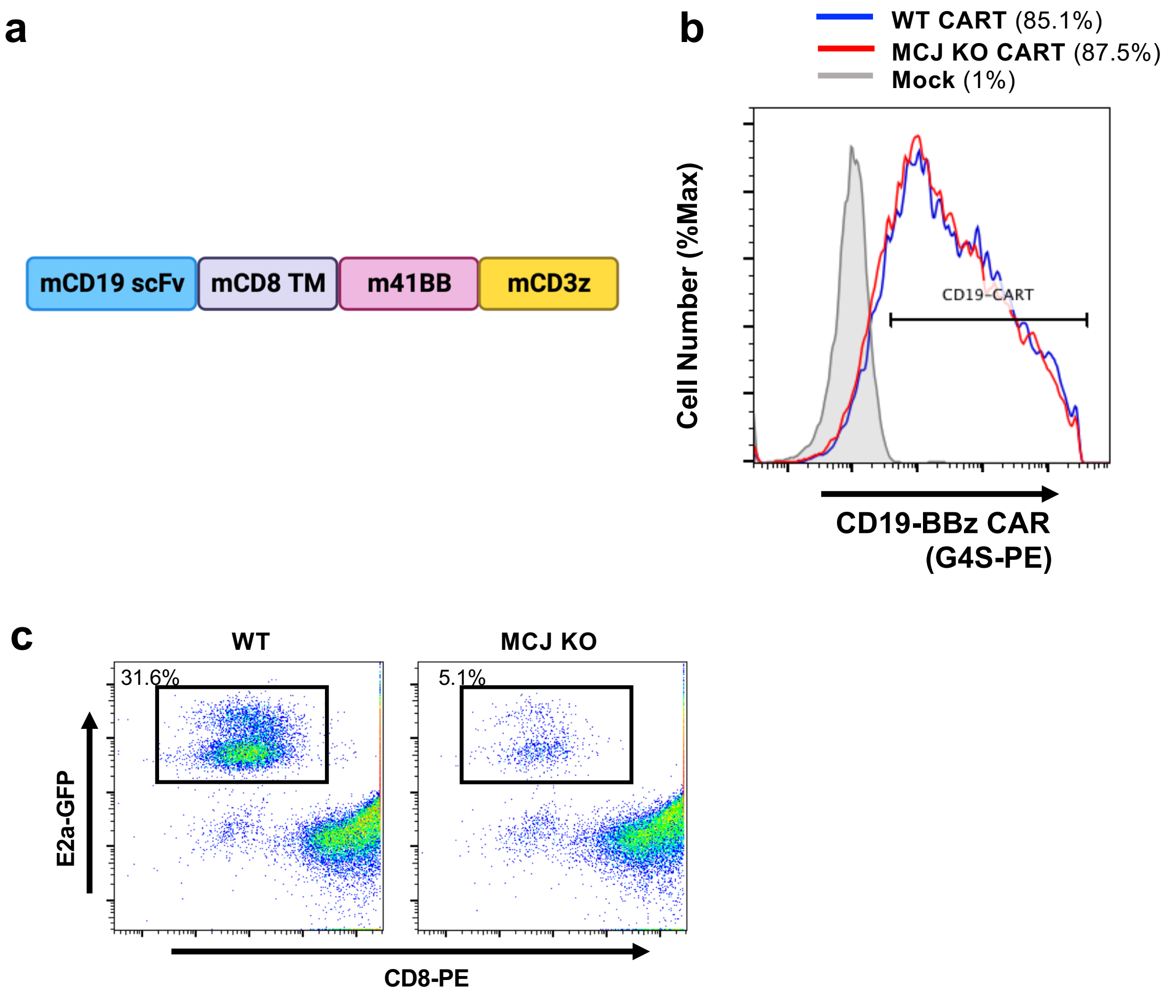

Supplementary Fig. 5: Expression of CAR on transduced mouse WT and MCJ KO CD8 CAR-T cells. (**a**) Scheme showing the murine CD19-BBz CAR construct containing a) the scFv part of mouse CD19 (mCD19 scFv), b) the transmembrane domain TM of mouse CD8 (mCD8 TM), c) the cytoplasmic costimulatory domain of mouse 4-1BB (m41BB), and d) the cytoplasmic domain of mouse CD3ζ (mCD3z). Created with BioRender.com (**b**) WT and MCJ KO CD8 cells were activated as in Fig. 3a, transduced with the CD19-BBz CAR retrovirus, expanded with IL-2 for 3 expansions, and expression of CD19-BBz CAR was examined by flow cytometry with a specific Ab against G4S linker. Percentage of positive population is shown. (**c**) The representative flow plots of the killing assay in Fig. 3b.

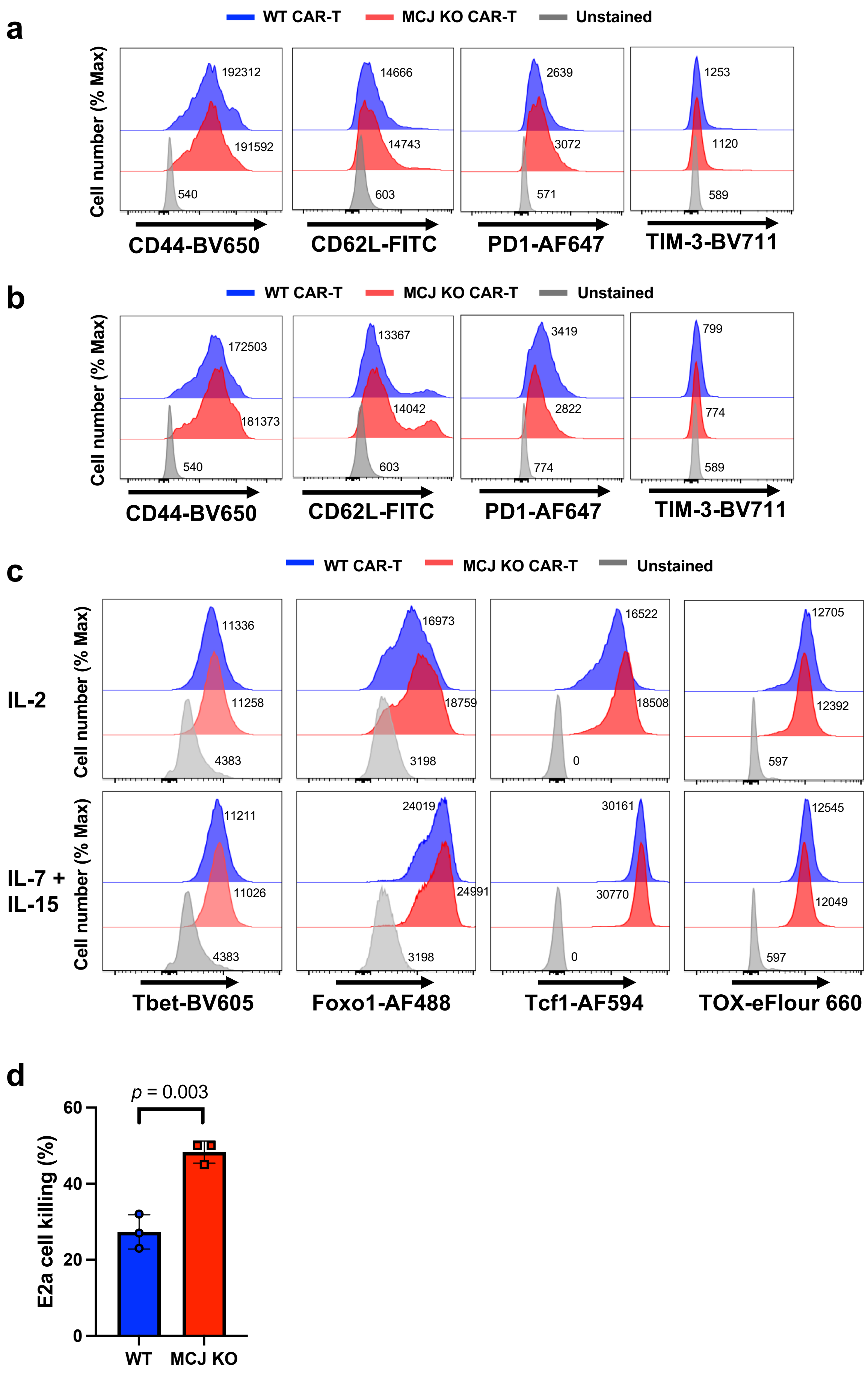

a
WT CAR-T
MCJ KO CAR-T
Unstained
Cell number (% Max)
192312
191592
540
14666
14743
603
2639
3072
571
1253
1120
589
CD44-BV650
CD62L-FITC
PD1-AF647
TIM-3-BV711
b
WT CAR-T
MCJ KO CAR-T
Unstained
Cell number (% Max)
172503
181373
540
13367
14042
603
3419
2822
774
799
774
589
CD44-BV650
CD62L-FITC
PD1-AF647
TIM-3-BV711
c
WT CAR-T
MCJ KO CAR-T
Unstained
IL-2
IL-7 +
IL-15
Cell number (% Max)
11336
11258
4383
16973
18759
3198
16522
18508
0
12705
12392
597
11211
11026
4383
24019
24991
3198
30161
30770
0
12545
12049
597
Tbet-BV605
Foxo1-AF488
Tcf1-AF594
TOX-eFlour 660
d
p = 0.003
E2a cell killing (%)
60
40
20
0
WT
MCJ KO

Supplementary Fig. 6: Expression of activation/differentiation markers in WT and MCJ KO CD19-BBz CAR-T cells. (**a**) WT and MCJ KO CD19-BBz CAR-T cells were generated by activation with anti-CD3/anti-CD28 Abs for 1 day, retrovirally transduced with CD19-BBz CAR, expanded with IL-2 (60 IU/ml) for 3 expansions, and rest in the medium for 24h. The surface activation markers (CD44, CD62L, PD-1, Tim3) expression were examined by flow cytometry. MFI is shown. (**b**) WT and MCJ KO CD19-BBz CAR-T cells were generated as in (a), washed, expanded in IL-7 (10 ng/ml) and IL-15 (100 ng/ml) for 3 expansions, and rested in the medium for 24h. The expression of activation markers (CD44, CD62L, PD-1, Tim3) were examined by flow cytometry. MFI is shown. (**c**) WT and MCJ KO CD19-BBz CAR-T cells were expanded with either IL-2 or IL-7/IL-15 for 3 expansions and cultured in medium for 24h as in (b). The transcription factor profiles were examined by intracellular staining and flow cytometry. MFI is shown. (**d**) WT and MCJ KO CD19-BBz CAR-T cells were generated and expanded with IL-7 and IL-15 for 3 expansions and rested in the medium for 24h as described in (b). The rested CAR-T cells were co-cultured with E2a cells for killing assay at E:T=0.5 (n=3 biologically independent cells). *p* was determined by two-sided unpaired t-test (**d**). Mean±SD is shown (**d**).

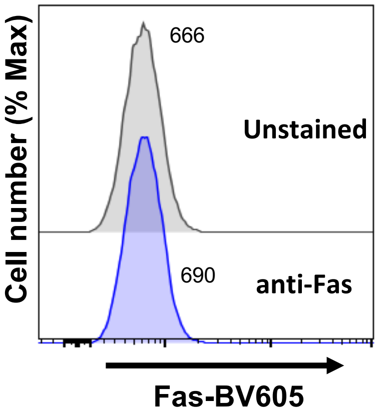

Supplementary Fig. 7: E2a cells do not express Fas on the cell surface. Fas expression on E2a target cells measured by flow cytometry. MFI is shown.

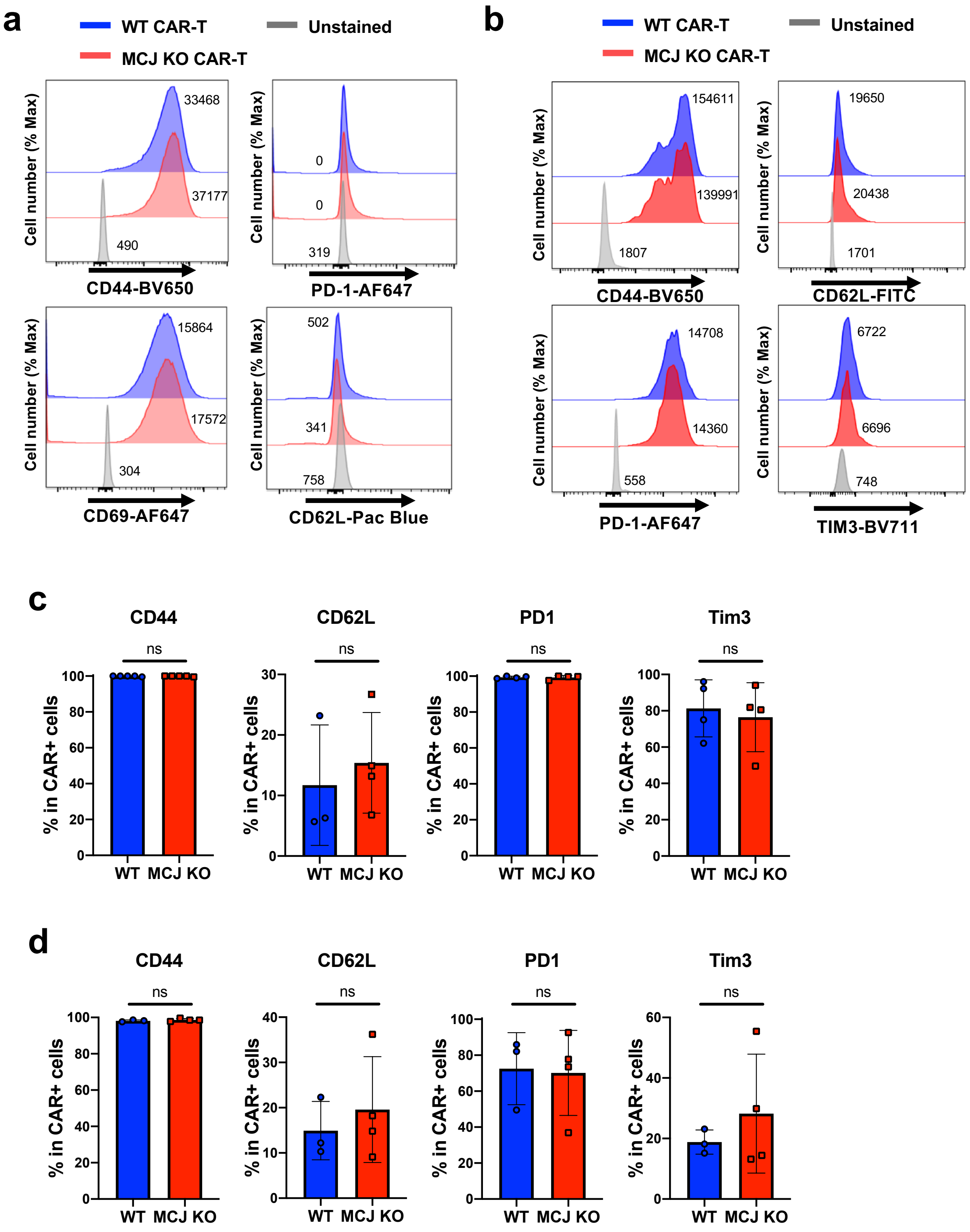
a
WT CAR-T
Unstained
MCJ KO CAR-T
Cell number (% Max)
33468
37177
490
CD44-BV650
0
0
319
PD-1-AF647
15864
17572
304
CD69-AF647
502
341
758
CD62L-Pac Blue
b
WT CAR-T
Unstained
MCJ KO CAR-T
154611
139991
1807
CD44-BV650
19650
20438
1701
CD62L-FITC
14708
14360
558
PD-1-AF647
6722
6696
748
TIM3-BV711
c
CD44
CD62L
PD1
Tim3
ns
% in CAR+ cells
WT
MCJ KO
d
CD44
CD62L
PD1
Tim3
ns
% in CAR+ cells
WT
MCJ KO

Supplementary Fig. 8: Expression of activation markers in WT and MCJ KO CD19-BBz CAR-T cells *in vivo*. (**a**) WT and MCJ KO CD8 cells were generated to express CD19-BBz CAR, and expanded with IL-2 (60 IU/ml) for 3 expansions. Activation markers (CD44, PD1, CD69 and CD62L) expression in the CD19-BBz CAR-T cells was examined by flow cytometry prior to being adoptively transferred to mice with leukemia (d=0). MFI is shown. (**b-d**) WT host were administrated with E2a cells (10^6 cells/mouse), irradiated, and treated with WT and MCJ KO CD19-BBz CAR-T cells (10^6 cells/mouse). After 7 days (n=5 mice, pooled) (**b**), 21 days (n=4 mice) (**c**) and 28 days (WT: n=3 mice, MCJ KO: n=4 mice) (**d**) post-CAR-T infusion, bone marrow of the CAR-T-treated mice were harvested, and stained for activation markers on CAR-T cells. MFI is shown for (b). "ns" denotes "not significant" as determined by $p>0.05$ by two-sided unpaired t test (**c, d**). Mean±SD is shown (**c, d**).

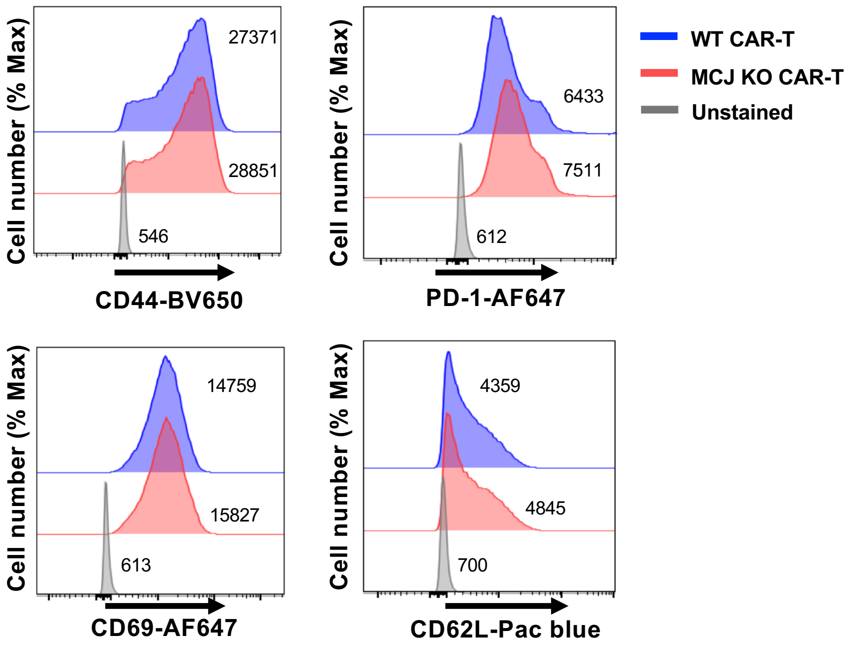

Supplementary Fig. 9: No difference in activation profiles of WT and MCJ KO CD19-BBz CAR-T cells expanded in IL-2 for 1 expansion prior to in vivo transfer. WT (blue) and MCJ KO (red) CD8 cells were activated and transduced with the CD19-BBz CAR as described in. 3aand grown with IL-2 (60 IU/ml) for 1 expansion. Activation markers (CD44, PD1, CD69, and CD62L) expression in the CD19-BBz CAR-T cells was examined by flow cytometry prior to being adoptively transferred to mice with leukemia. Histograms of unstained cells are shown (gray). MFI is shown.

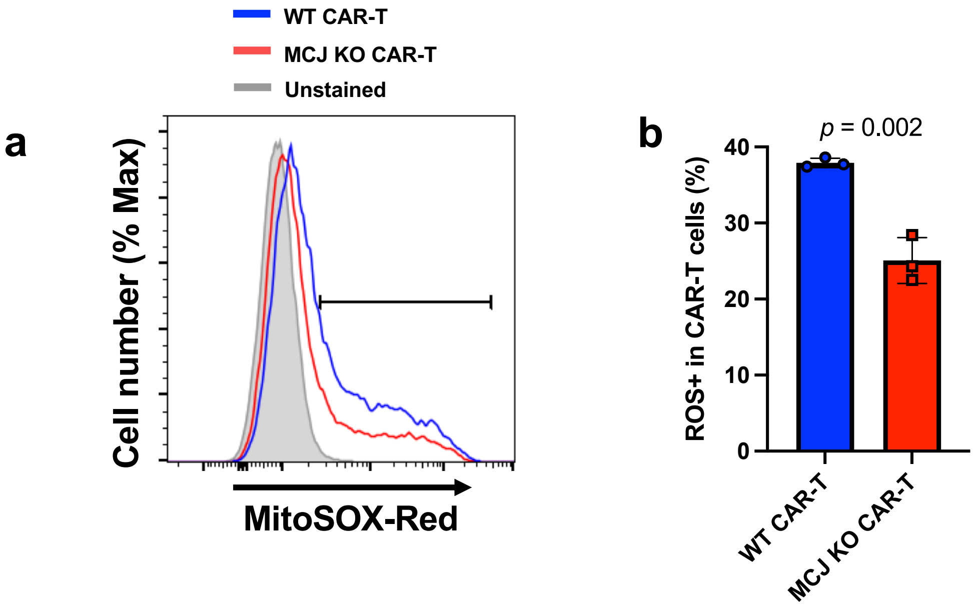

Supplementary Fig. 10: Reduced ROS production in MCJ KO CD19-BBz CAR-T cells. WT and MCJ KO CD8 cells were generated to express CD19-BBz CAR and expanded with IL-2 for 3 expansions. Mitochondrrial ROS in the CD19-BBz CAR-T cells was examined by flow cytometry using MitoSOX Red staining and flow cytometry. (**a**) Representative profile. (**b**) Percentage of ROS+ CAR-T cells between 2 groups (n=3 replicate cells). The positive population is gated in (a). *p* was determined by two-sided unpaired t test (**b**). Mean±SD is shown (**b**).

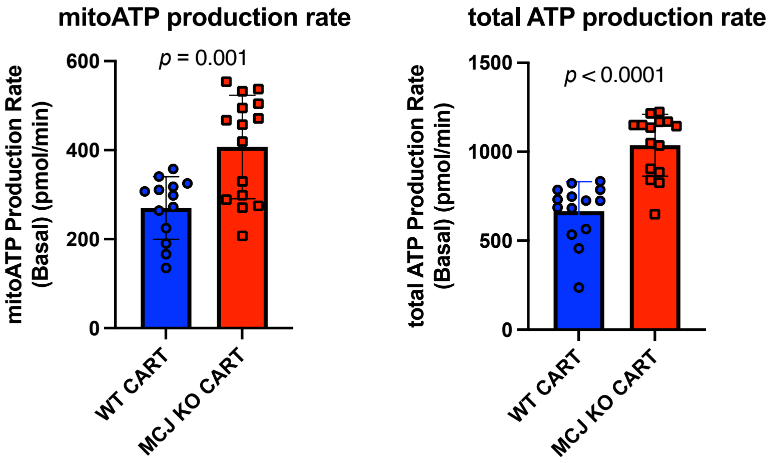

Supplementary Fig. 11: Increased ATP production in MCJ KO CD19-BBz CAR-T cells. WT and MCJ KO CD8 cells were activated, transduced to express CD19-BBz CAR and expanded with IL-2 for 3 expansions. The CD19-BBz CAR-T cells were isolated and used for seahorse ATP real-time rate assay (n=14 replicate wells). The total ATP production rate and ATP production rate from mitochondria are calculated using the ATP rate analysis in Wave. *p* was determined by two-sided unpaired t-test. Mean±SD is shown.

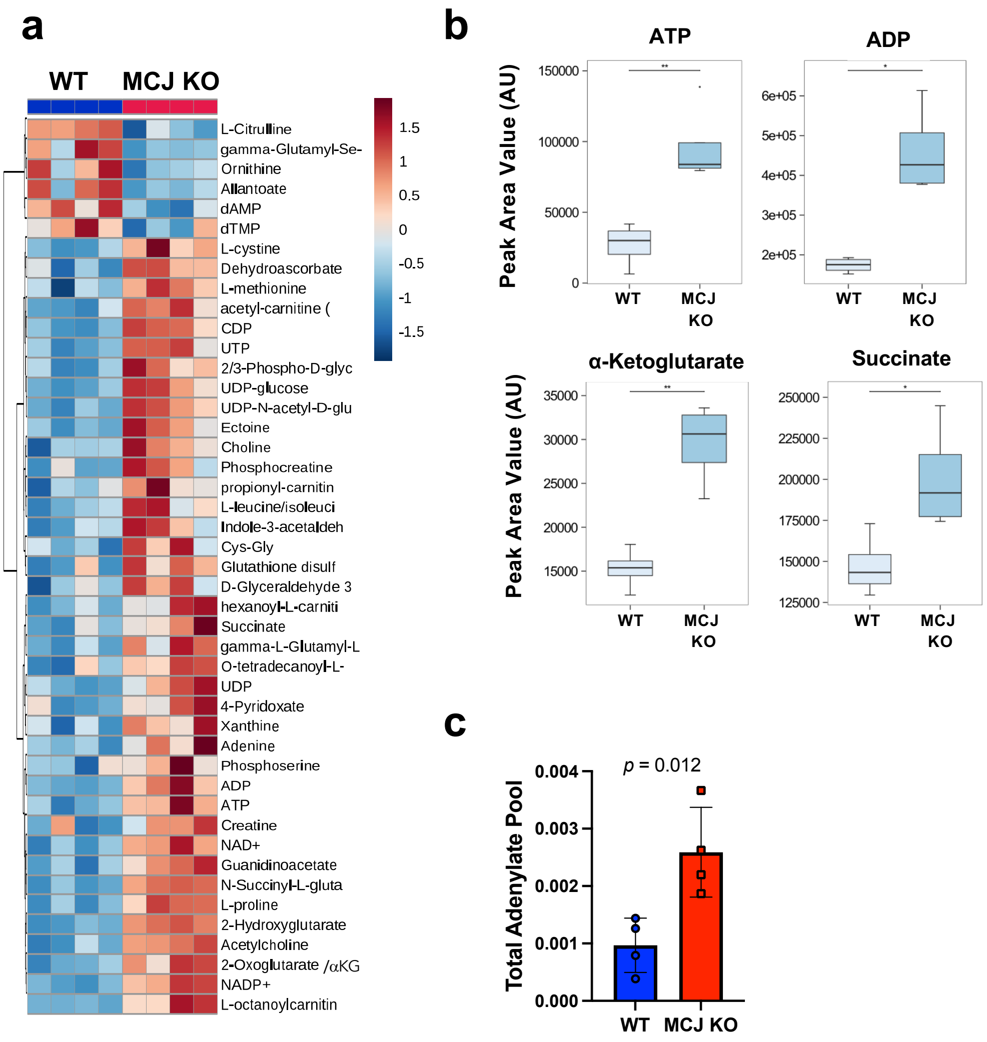

Supplementary Fig. 12: Increased metabolites involved in mitochondrial respiration in MCJ KO CD8 CAR-T cells. (**a**) Hierarchical clustering analysis of the top 50 metabolites by T-test analysis of relative abundances in purified WT and MCJ KO CD8 CAR-T cells upon three expansions with IL-2 (n=4 replicate cells). (**b**) Peak area values (AU, arbitrary unit) of some of the most significant metabolites that emerged from this analysis, with a focus on high energy purine phosphate nucleosides (ATP and ADP) and mitochondrial metabolites (alpha-ketoglutarate and succinate) that are affected by MCJ KO in CD8 CAR-T cells (n=4). (**c**) Total adenylate pool of two groups calculated based on the formula: (ATP+ 0.5xADP)/(ATP+ADP+AMP). *p* was determined by two-sided unpaired t-test. Mean±SD is shown (**c**).

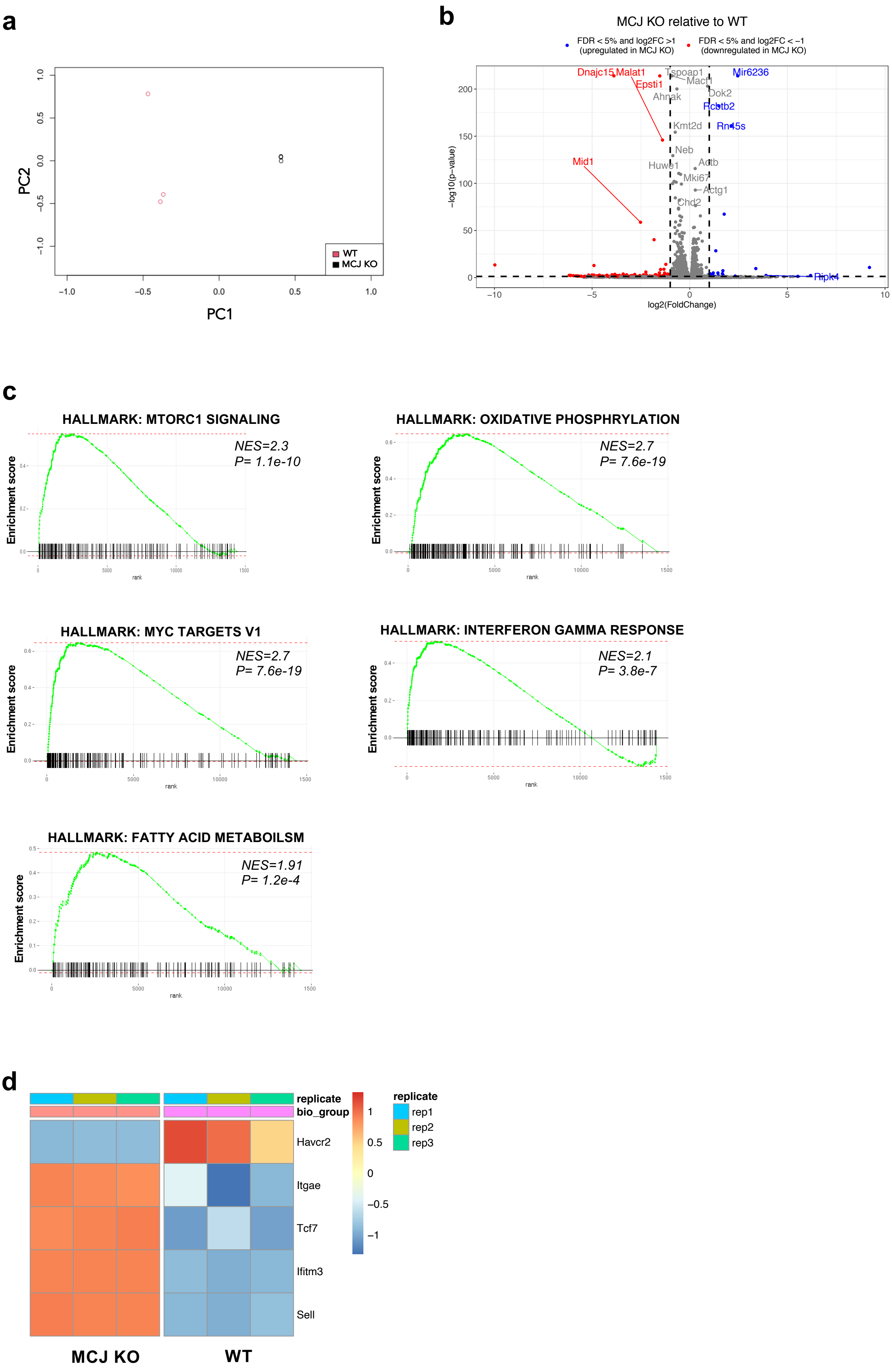
a
PC1
PC2
WT
MCJ KO
b
MCJ KO relative to WT
FDR < 5% and log2FC >1
(upregulated in MCJ KO)
FDR < 5% and log2FC < −1
(downregulated in MCJ KO)
−log10(p−value)
log2(FoldChange)
Dnajc15
Malat1
Tspoap1
Macf1
Mir6236
Epsti1
Dok2
Ahnak
Rcbtb2
Kmt2d
Rn45s
Neb
Mid1
Huwe1
Actb
Mki67
Actg1
Chd2
Ripk4
c
HALLMARK: MTORC1 SIGNALING
NES=2.3
P= 1.1e-10
HALLMARK: OXIDATIVE PHOSPHRYLATION
NES=2.7
P= 7.6e-19
HALLMARK: MYC TARGETS V1
NES=2.7
P= 7.6e-19
HALLMARK: INTERFERON GAMMA RESPONSE
NES=2.1
P= 3.8e-7
HALLMARK: FATTY ACID METABOILSM
NES=1.91
P= 1.2e-4
Enrichment score
rank
d
replicate
bio_group
Havcr2
Itgae
Tcf7
Ifitm3
Sell
rep1
rep2
rep3
MCJ KO
WT

Supplementary Fig. 13: RNAseq analysis between MCJ KO CD19-BBz CAR-T cells and WT CD19-BBz CAR-T cells. RNAseq analyses of WT and MCJ KO CD19-BBz CAR-T cells after 3 expansions with IL-2 (n=3 replicate cells). (**a**) Principal component analysis of 2 groups after correcting for covariates. (**b**) Volcano plot illustrating all genes analyzed in differential expression analysis between 2 groups. p-value is shown. (**c**) GSEA plots of top enriched pathways of MCJ KO CAR-T cells compared to WT CAR-T cells. (**d**) Heatmap of transcripts difference for selective genes related to T cell differentiation. NES = normalized enrichment score. Adjusted p-value is shown and calculated using Benjamini-Hochberg procedure using 10,000 permutations.

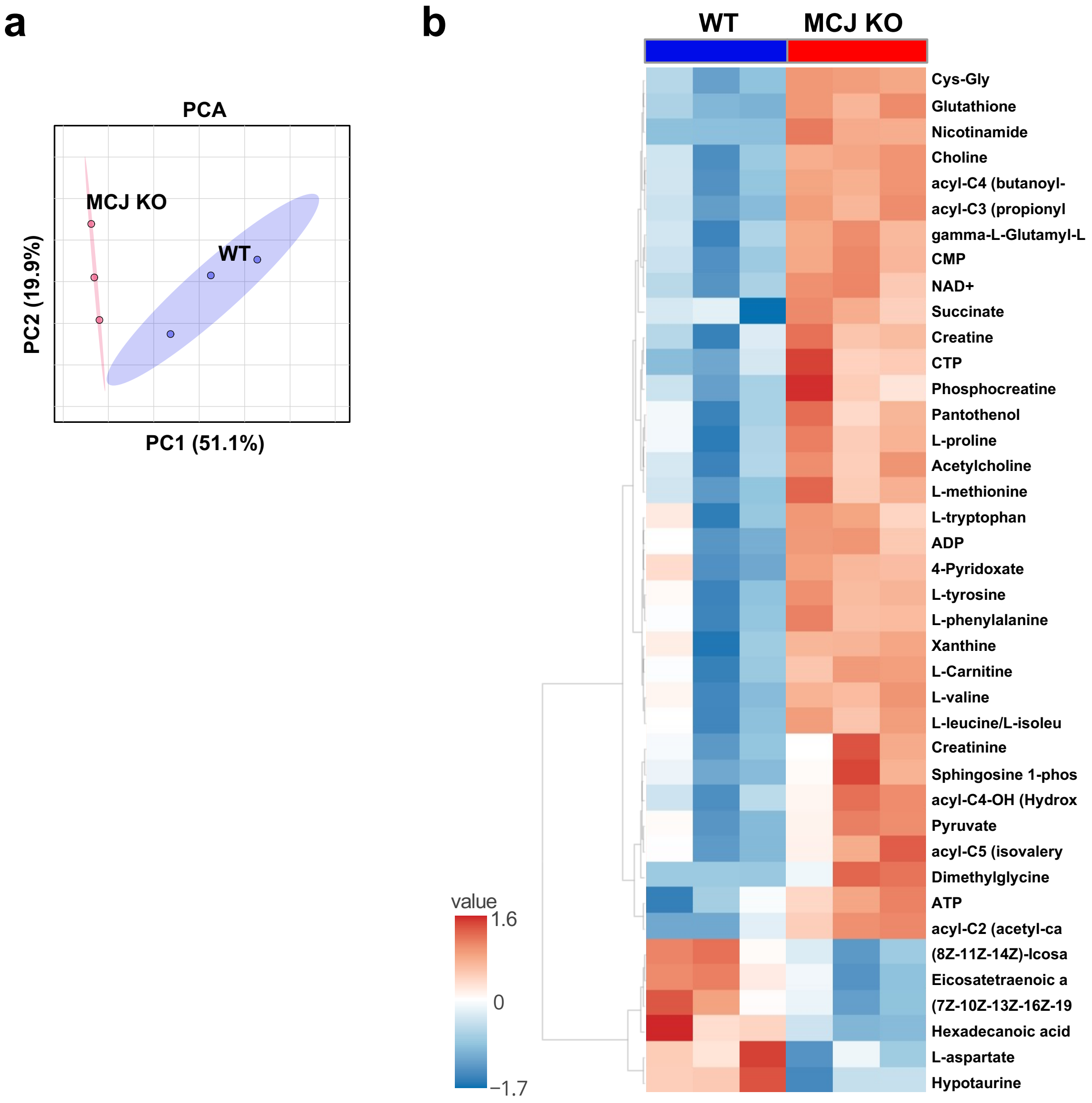

Supplementary Fig. 14: Metabolomic profile of WT and MCJ KO CD8 CAR-T cells after 3 expansions followed by cytokine-free medium. Mass spectrometry-based metabolomics analysis of purified WT and MCJ KO CD8 CAR-T cells after three expansions with IL-2 and rest in the medium for 48h (n=3 replicate cells). (**a**) Principal component analysis of WT and MCJ KO CAR-T cells. (**b**) Hierarchical clustering analysis of the top 40 metabolites of relative abundances in purified WT and MCJ KO CD8 CAR-T cells.

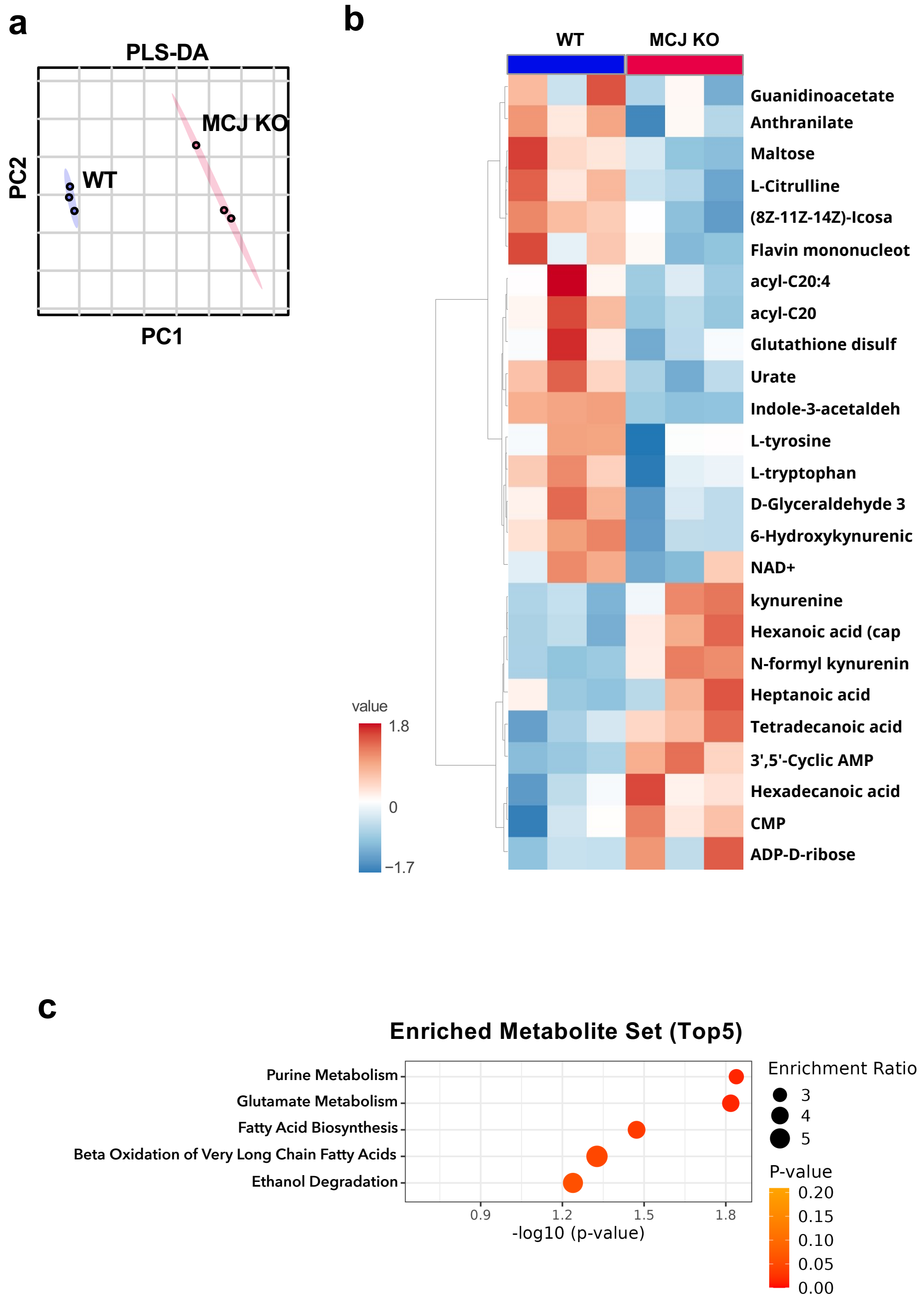

Supplementary Fig. 15: Metabolomic profile of in vivo WT and MCJ KO CD8 CAR-T cells. WT host were administrated with E2a cells (10^6 cells/mouse), irradiated, and infused with WT and MCJ KO CD19-BBz CAR-T cells ($2x10^6$ cells/mouse) as described in Fig. 4a. After 4 days of CAR-T cells infusion, the bone marrows of CAR-T treated mice were harvested. The CAR-T cells were enriched, pooled and collected for metabolomics analysis (n=3 replicate cells pooled from 3 mice). (**a**) PLS-DA analysis between two groups. (**b**) Hierarchical clustering analysis of the top 25 metabolites in purified WT and MCJ KO CD8 CAR-T cells harvested from mice. (**c**) Enrichment pathway analysis of MCJ KO CD19-BBz CAR-T cells compared to WT CAR-T cells based on top 40 distinct metabolites.

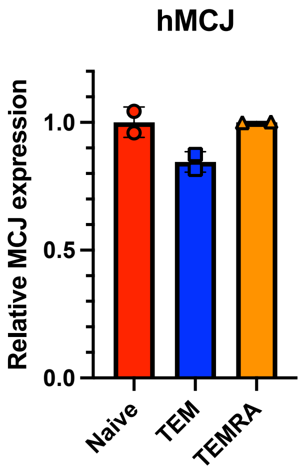

Supplementary Fig. 16: Expression MCJ in different human CD8 cell subtypes. Human CD8 cells from PBMC were sorted for naïve ($CCR7^+/CD45RA^+/CD45RO^-$), effector memory (TEM) ($CCR7^-/CD45RA^-/CD45RO^+$), and effector memory T cells re-expressing CD45RA (TEMRA) ($CCR7^-/CD45RA^+/CD45RO^-$). MCJ expression was determined by real time RT-PCR (n=2 replicates per subtype). Fold induction relative to HPRT is shown. Mean±SD is shown.

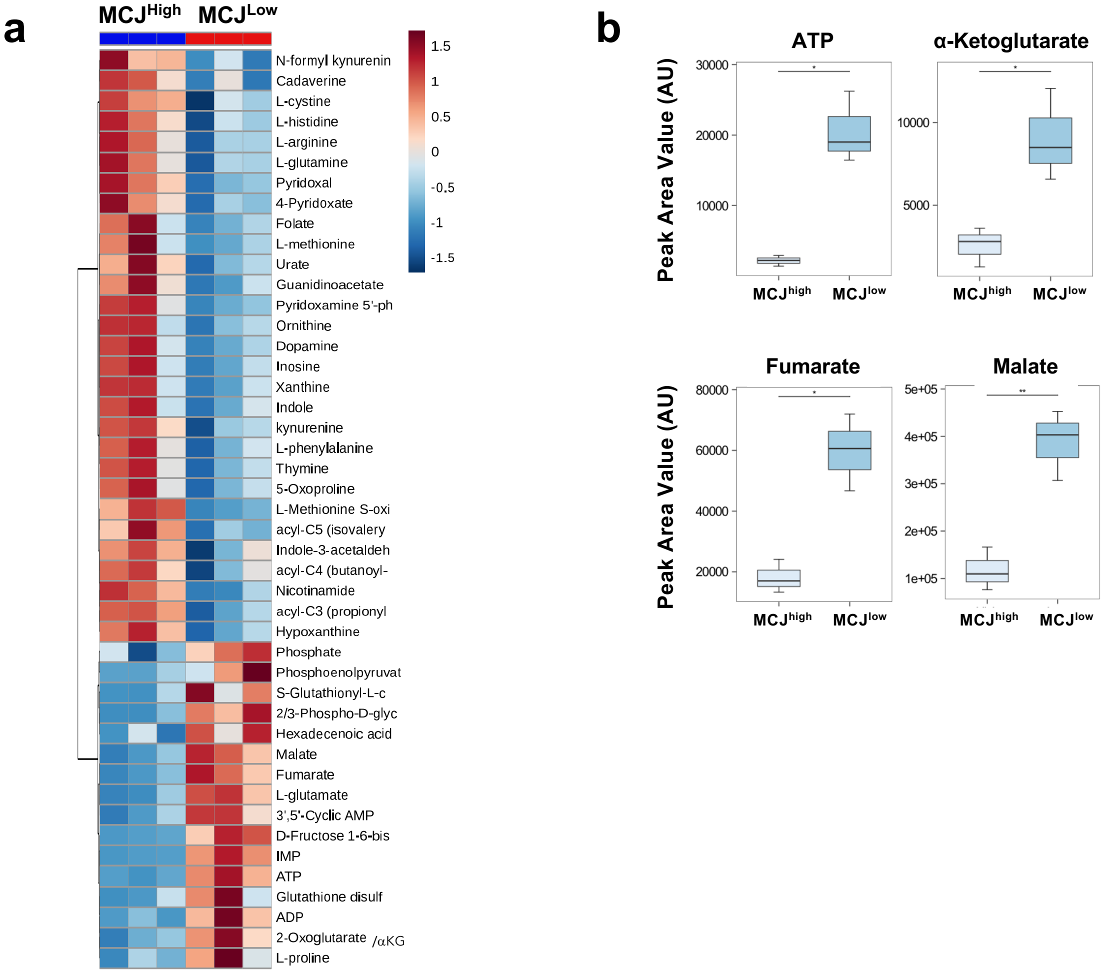

Supplementary Fig. 17: Increased metabolites involved in mitochondrial respiration in low MCJ-expressing human CD8 CAR-T cells. (**a**) Hierarchical clustering analysis of the top 45 metabolites by T-test analysis of relative abundances in D16 (MCJ^{low}) and D26 (MCJ^{high}) CAR-T cells (3 expansions) (n=3 replicate cells). (**b**) Peak area values (AU, arbitrary unit) of some of the most significant metabolites that emerged from this analysis, with a focus on high energy purine phosphate nucleosides (ATP) and mitochondrial metabolites (alpha-ketoglutarate, fumarate, and malate) that are affected by MCJ KO in CD8 CAR-T cells (n=3 replicate cells).

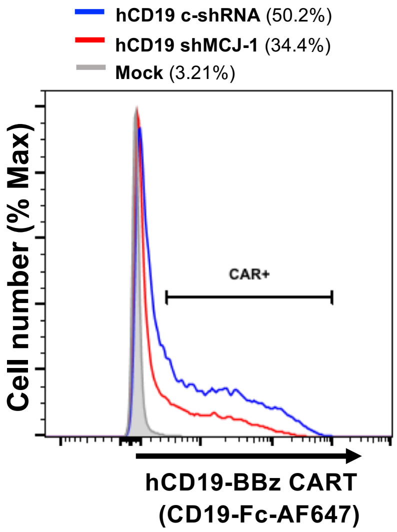

Supplementary Fig. 18: Expression of CAR on human CD8 cells transduced CD19-BBz/c-shRNA CAR and CD19-BBz/shMCJ CAR. Human CD8 cells were activated as described in Fig. 7d-g, transduced with either human CD19-BBz/c-shRNA CAR or CD19-BBz/shMCJ-1 CAR, expanded with IL-2 (100 IU/ml) for three expansions, and the expression of CAR was examined by flow cytometry using a CD19-Fc AF647 conjugated peptide. Percentage of positive population is shown.

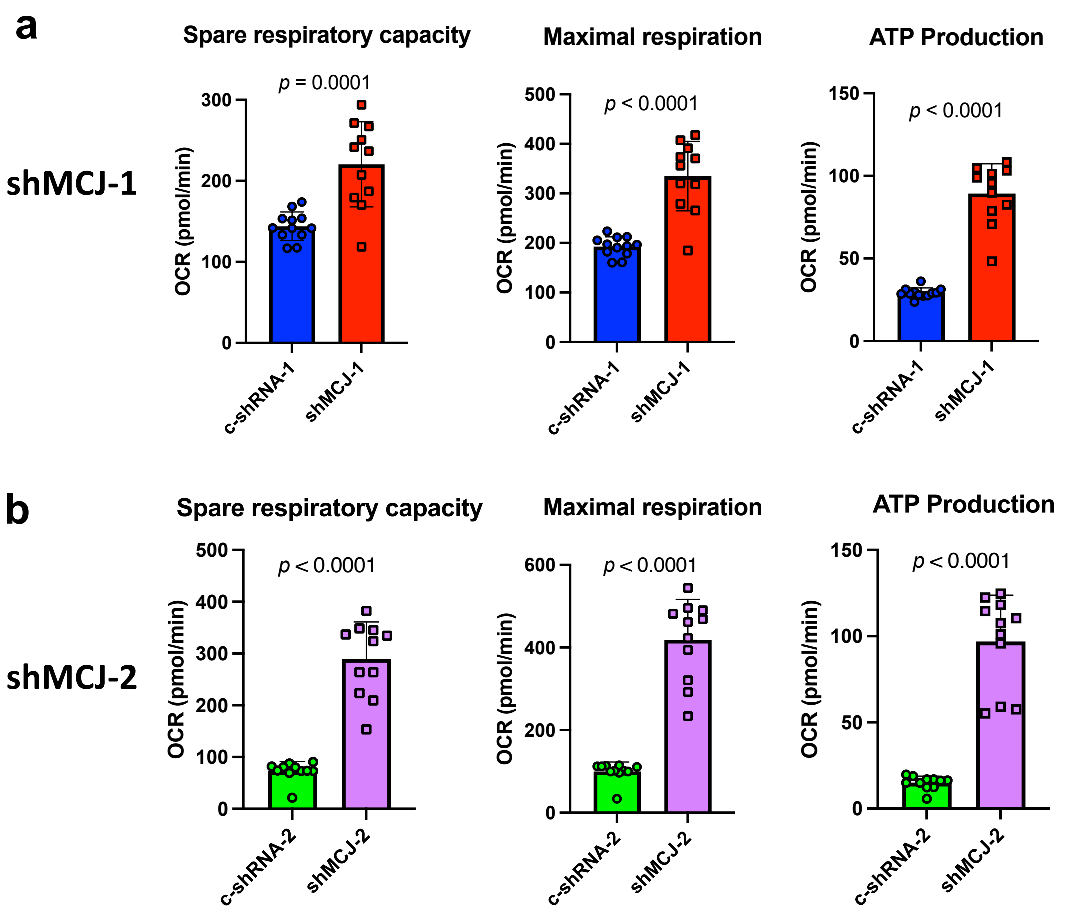

Supplementary Fig. 19: Increased mitochondrial respiration in human CD8 CAR-T cells by silencing MCJ expression. (**a-b**) CD8 cells from a MCJ^{high} donor were isolated, activated and transduced with CD19-BBz-c-shRNA-1 CAR or CD19-BBz-shMCJ-1 CAR vectors (**a**), or CD19-BBz-c-shRNA-2 CAR or CD19-BBz-shMCJ-2 CAR vectors (**b**). After 3 expansions with IL-2 (100 IU/ml), CAR+ cells were isolated and used for the Seahorse MitoStress assay. Spare respiratory capacity, maximal respiration, and ATP production were determined as recommended by the manufacturer (n=10 replicate wells). *p* was determined by two-sided unpaired t-test (**a, b**). Mean±SD is shown (**a, b**).

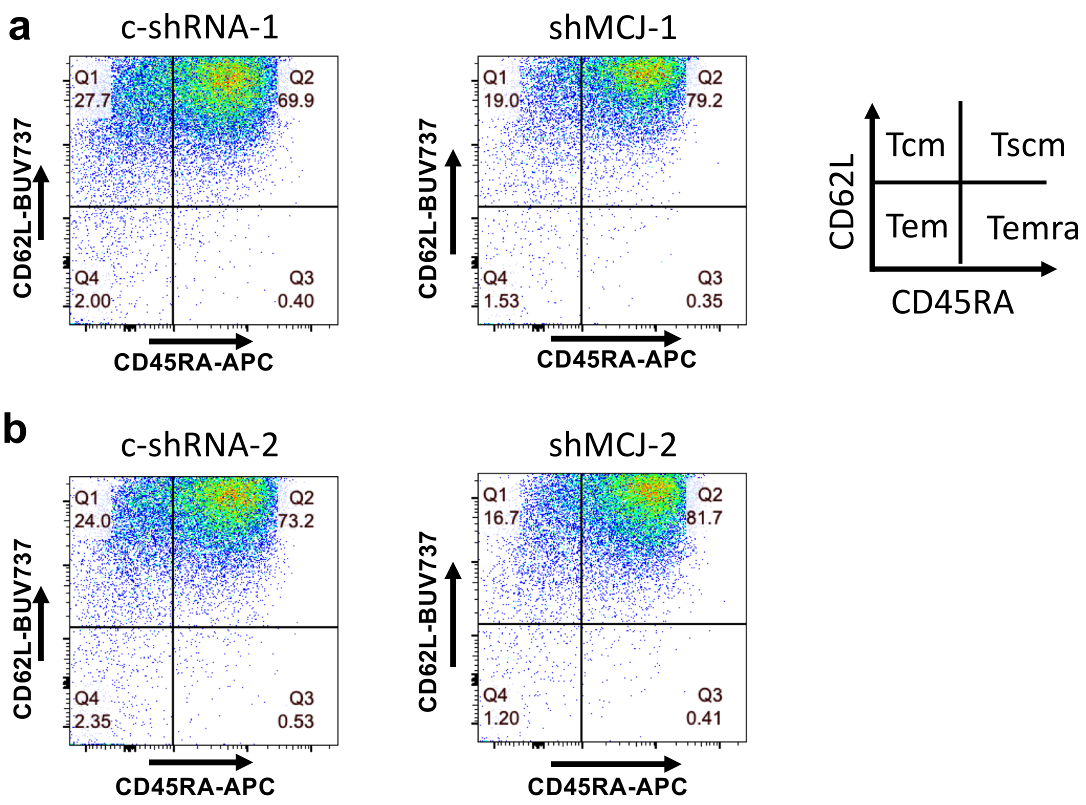

Supplementary Fig. 20: Subtypes of memory/effector cell in human CD19-BBz-shMCJ CD8 CAR T cells. (**a-b**) CD8 cells from a MCJ^{high} donor were isolated, activated and transduced with CD19-BBz-c-shRNA-1 CAR or CD19-BBz-shMCJ-1 CAR vectors (**a**) or CD19-BBz-c-shRNA-2 CAR or CD19-BBz-shMCJ-2 CAR vectors (**b**). After 3 expansions with IL-2 (100 IU/ml), the expression of CD45RA and CD62L were determined by immunostaining and flow cytometry.

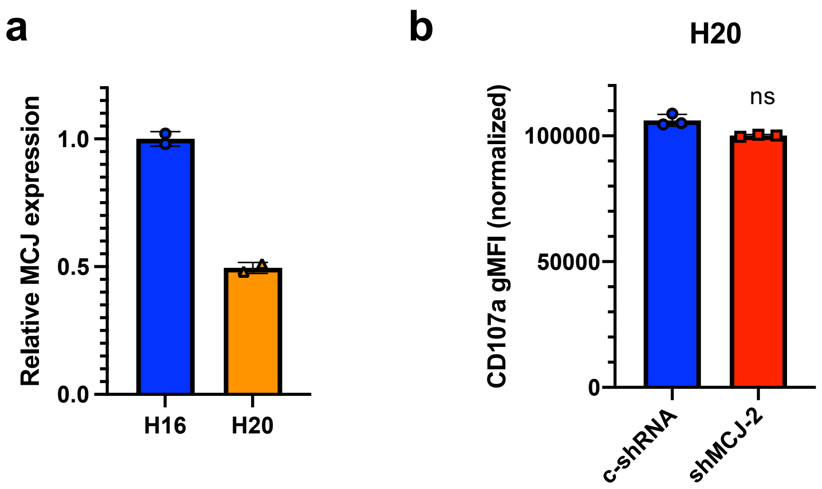

Supplementary Fig. 21: Effect of MCJ silencing in MCJ^{low} CD8 CAR-T cells. (**a**) MCJ expression in CD8 cells isolated from PBMC of donor H16 (MCJ^{high}) and H20 (MCJ^{low}) as determine by real time RT-PCR. Fold induction relative to HPRT is shown (n=2 replicates) (**b**) CD8 cells from donor H20 (MCJ^{low}) were isolated, activated and transduced with CD19-BBz-c-shRNA CAR or CD19-BBz-shMCJ-2 CAR vectors. The CAR-T cells were expanded in IL-2 (100 IU/ml) for 3 expansions, and co-cultured with Nalm6 cells for 4h to examine the cytotoxic activity by CD107a staining and flow cytometry analysis (n=3 biologically independent cells). “ns” denotes “not significant” as determined by $p>0.05$ by unpaired Mann-Whitney test (**b**). Mean±SD is shown (**a, b**).

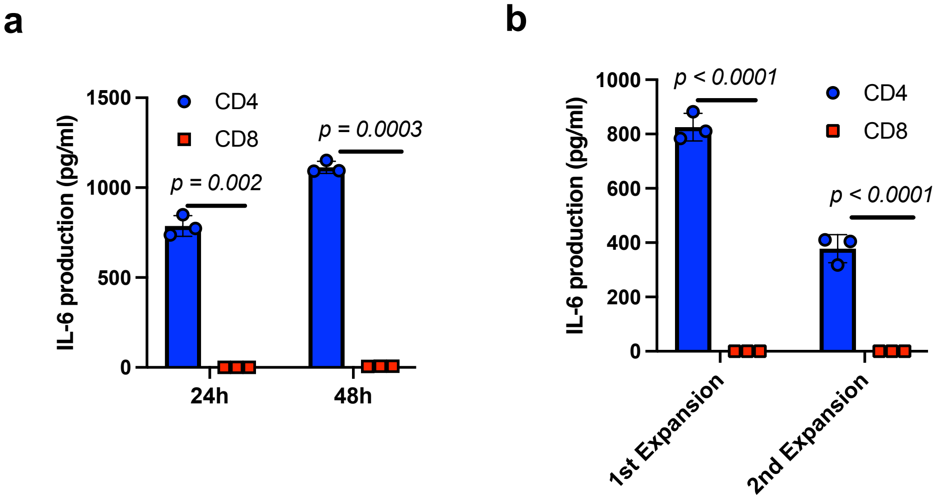

Supplementary Fig. 22: Production of IL-6 by human CD4 cells, but not human CD8 cells. (**a**) Human CD4 and CD8 cells were isolated from PBMC of a healthy donor and activated with anti-CD3 and anti-CD28 Abs for 48h. IL-6 levels in the supernatant was determined in by ELISA (n=3 biologically independent samples). (**b**) CD4 and CD8 cells were isolated from PBMC, activated as described in (a), and expanded with IL-2 (100 IU/ml) for 1 or 2 expansions. IL-6 in the supernatant in cells grown with IL-2 alone was measured by ELISA (n=3 biologically independent samples). *p* was determined by unpaired t-test (**a, b**). Mean±SD is shown (**a, b**).

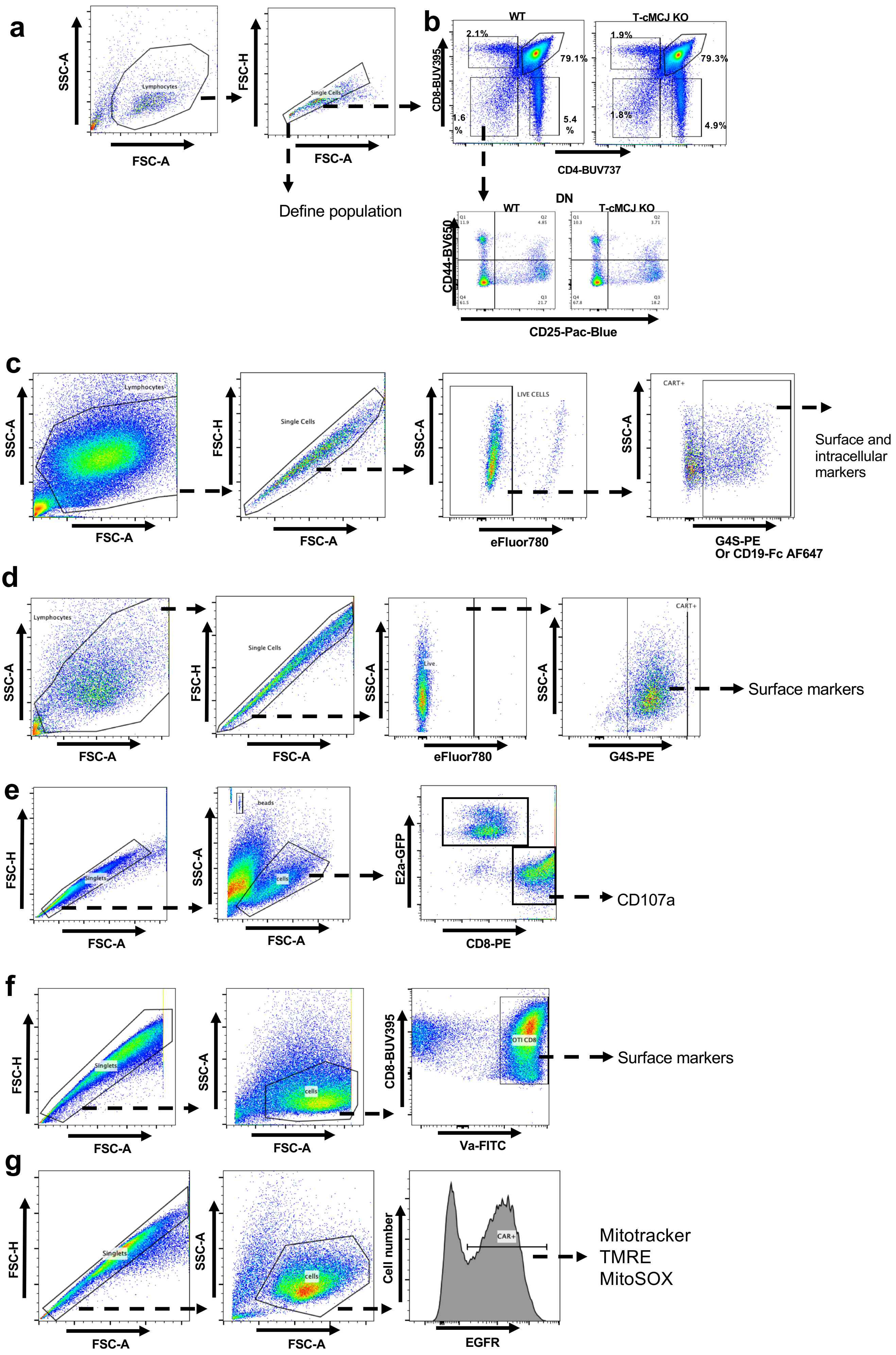

a
SSC-A
FSC-A
Lymphocytes
FSC-H
Single Cells
Define population
b
WT
T-cMCJ KO
CD8-BUV395
CD4-BUV737
2.1%
79.1%
1.6 %
5.4 %
1.9%
79.3%
1.8%
4.9%
DN
CD44-BV650
CD25-Pac-Blue
c
LIVE CELLS
eFluor780
CART+
G4S-PE
Or CD19-Fc AF647
Surface and intracellular markers
d
Live
Surface markers
e
Singlets
beads
cells
E2a-GFP
CD8-PE
CD107a
f
CD8-BUV395
OTI CD8
Va-FITC
g
Cell number
CAR+
EGFR
Mitotracker
TMRE
MitoSOX

Supplementary Fig. 23: Gating Strategies. (**a**) Gating strategies for Fig. 6b&f, Fig. 7b, Supp. Fig. 1d-g, Supp. Fig. 5b, Supp. Fig. 7, Supp. Fig. 18. (**b**) Gating strategies for defining thymus population in Supp. Fig. 1b-c. (**c**) Gating strategies for surface and intracellular markers staining in Supp. Fig. 6 and Supp. Fig. 20. (**d**) Gating strategies for surface marker staining in Supp. Fig. 8 & Supp. Fig. 9. (**e**) Gating strategies for killing assay in Supp. Fig. 5c, and CD107a assay in Fig. 3g. (**f**) Gating strategies for surface marker staining in Supp. Fig. 4. (**g**) Gating strategies for Fig. 5a-d and Supp. Fig. 10.

Supplementary Table I

antibody	conjugate	catalog #	manufacturer	clone	dilutiion	validation	website link
anti-human EGFR	PE	352904	BioLegend	AY13	1 in 200	Human cervical cancer cell line HELA was stained with EGFR (clone AY13) PE (filled histogram) or mouse IgG1, κ PE isotype control (open histogram). Citation: Lu T, et al. 2022. Nat Commun. 13:2576.	https://www.biolegend.com/de-at/products/pe-anti-human-egfr-antibody-7432
anti-human EGFR	APC	352906	BioLegend	AY14	1 in 200	Human cervical cancer cell line HELA was stained with EGFR (clone AY13) APC (filled histogram) or mouse IgG1, κ APC isotype control (open histogram). Citation: Dong C, et al. 2022. Nat Commun. 13:2548	https://www.biolegend.com/de-at/products/apc-anti-human-egfr-antibody-7714
anti-β-actin		4970S	Cell Signaling Technology	13E5	1in 1000	Western blot analysis of cell extracts from various cell lines using beta-Actin (13E5) Rabbit mAb. The correct band size is shown	https://www.cellsignal.com/products/primary-antibodies/b-actin-13e5-rabbit-mab/4970
anti-G4S linker	PE	38907S	Cell Signaling Technology	E7O2V	1 in 200	Flow cytometric analysis of live pan-CD3+ T cells isolated from human PBMCs and engineered to express an scFv-based Anti-CD20 CAR containing a G4S linker, using G4S Linker (E7O2V) Rabbit mAb (PE Conjugate) (right) or concentration-matched Rabbit (DA1E) mAb IgG XP® Isotype Control (PE Conjugate) #5742 (left). Tag Blue fluorescent protein (TagBFP) is co-expressed with the CAR. Data courtesy of Michael Kvorjak, Lohmueller lab (University of Pittsburgh).	https://www.cellsignal.com/products/antibody-conjugates/g4s-linker-e7o2v-rabbit-mab-pe-conjugate/38907
anti-GAPDH		2118S	Cell Signaling Technology	14C10	1in 2500	Western blot analysis of extracts from various cell lines using GAPDH (14C10) Rabbit mAb. The correct band is shown	https://www.cellsignal.com/products/primary-antibodies/gapdh-14c10-rabbit-mab/2118
anti-NDUFA9		459100	Thermo Fisher Scientific Invitrogen	20C11B11B11		This Antibody was verified by Knockdown to ensure that the antibody binds to the antigen stated. Western blot was performed using Anti-NDUFA9 Monoclonal Antibody (20C11B11B11) (Product # 45-9100) and a 36 kDa band corresponding to NDUFA9 was observed across cell lines and tissues tested.	https://www.thermofisher.com/antibody/product/NDUFA9-Antibody-clone-20C11B11B11-Monoclonal/459100
anti-human CCR7	PE	353204	BioLegend	G043H7	1 in 200	Human peripheral blood lymphocytes were stained with CD3 APC/Cy7 and CCR7/CD197 (clone G043H7) PE (top) or mouse IgG2a, κ PE isotype control (bottom). Citation: Kramer KJ, et al. 2022. Nat Commun. 13:3466.	https://www.biolegend.com/nl-nl/products/pe-anti-human-cd197-ccr7-antibody-7498
anti-human CD107a	BV605	328634	BioLegend	H4A3	1 in 100	Thrombin-activated human peripheral blood platelets were stained with CD107a (clone H4A3) Brilliant Violet 605™ (filled histogram) or mouse IgG1, κ Brilliant Violet 605™ isotype control (open histogram). Citation: Labanieh L, et al. 2022. Cell. 185:1745.	https://www.biolegend.com/nl-be/sean-tuckers-tests/brilliant-violet-605-anti-human-cd107a-lamp-1-antibody-8975?GroupID=BLG10252
anti-human CD28		302933	BioLegend	CD28.2	4ug/ml	Human peripheral blood lymphocytes stained with LEAF™ purified CD28.2, followed by anti-mouse IgGs FITC. Citation: Pozzetto B, et al. 2021. Nature. 600:701.	https://www.biolegend.com/de-at/products/ultra-leaf-purified-anti-human-cd28-antibody-7743
anti-human CD3		300414	BioLegend	UCHT1	10ug/ml	Human peripheral blood lymphocytes stained with purified UCHT1 and then detected with anti-mouse IgGs FITC. Citation: Dallari S, et al. 2017. Nat Commun. 8:14830.	https://www.biolegend.com/ja-jp/products/purified-anti-human-cd3-antibody-867
anti-human CD4	Alexa Fluor 647	317422	BioLegend	OKT4	1 in 200	Human peripheral blood lymphocytes stained with OKT4 Alexa Fluor® 647. Citation: Greenwood EJD et al. 2019. Cell Rep. 27(5):1579-1596	https://www.biolegend.com/en-ie/products/alexa-fluor-647-anti-human-cd4-antibody-3660
anti-human CD45RA	Alexa Fluor 700	304120	BioLegend	HI100	1 in 200	Human peripheral blood lymphocytes stained with HI100 Alexa Fluor® 700. Citation: Evans RDR, et al. 2020. Nat Commun. 3.491666667	https://www.biolegend.com/nl-be/products/alexa-fluor-700-anti-human-cd45ra-antibody-3421
anti-human CD45RA	APC	550855	BD Biosciences	HI100	1 in 40	Multicolor flow cytometric analysis of CD45RA expression on human peripheral blood lymphocytes. Human whole blood was stained with PE Mouse Anti-Human CD45RO antibody (Cat. No. 555493/561889), and with either APC Mouse IgG2b, κ Isotype Control (Cat. No. 555745; Left Plot) or APC Mouse Anti-Human CD45RA antibody (Cat. No. 550855/561884; Right Plot). The erythrocytes were lysed with BD FACS™ Lysing Solution (Cat. No. 349202). Bivariate pseudocolor density plots showing the correlated expression of CD45RA (or Ig Isotype control) versus CD45RO was derived from gated events with the forward and side light-scatter characteristics of intact lymphocytes. Flow cytometry and data analysis were performed using a BD LSRFortessa™ Cell Analyzer System and FlowJo™ software.	https://www.bdbiosciences.com/en-us/products/reagents/flow-cytometry-reagents/research-reagents/single-color-antibodies-ruo/apc-mouse-anti-human-cd45ra.561884
anti-human CD45RO	FITC	304204	BioLegend	UCHL1	1 in 200	Human peripheral blood lymphocytes stained with UCHL1 FITC. Citation: Fang F, et al. 2021. Cell Rep. 37:109981.	https://www.biolegend.com/de-de/products/fitc-anti-human-cd45ro-antibody-857

Supplementary Table I

anti-human CD62L	BUV737	741843	BD Biosciences	DREG-56	1 in 200	Citation: Kishimoto TK et al. Blood. 78(3):805-811	https://www.bdbiosciences.com/en-eu/products/reagents/flow-cytometry-reagents/research-reagents/single-color-antibodies-ruo/buv737-mouse-anti-human-cd62l.741843
anti-human CD8a	Pacific Blue	300928	BioLegend	HIT8a	1 in 200	Human peripheral blood lymphocytes stained with HIT8a Pacific Blue™. Citation: Khatamzas E, et al. 2022. Nat Commun. 13:5586.	https://www.biolegend.com/de-de/products/pacific-blue-anti-human-cd8a-antibody-6659?GroupID=BLG5903
anti-human CD8a	BV785	301046	BioLegend	RPA-T8	1 in 200	Human peripheral blood lymphocytes were stained with CD3 FITC and CD8 (clone RPA-T8) Brilliant Violet 785™. Evans RDR, et al. 2020. Nat Commun. 3.491666667.	https://www.biolegend.com/en-gb/products/brilliant-violet-785-anti-human-cd8a-antibody-7919?GroupID=BLG5903
anti-human MCJ					1 in 333	It was verified in previous study, Hatle et al. Mol Cell Bio. 27:2952–2966.	
anti-mouse/human B220	FITC	103205	BioLegend	RA3-6B2	1 in 200	C57BL/6 mouse splenocytes stained with RA3-6B2 FITC. Citation: Zaman R, et al. 2021. Immunity.	https://www.biolegend.com/fr-lu/products/fitc-anti-mouse-human-cd45r-b220-antibody-445?GroupID=GROUP658
anti-mouse CD107a	Alexa Fluor 700	121628	BioLegend	1D4B	1 in 100	Thioglycollate-elicited BALB/c mouse peritoneal macrophages were stained with CD107a (clone 1D4B) Alexa Fluor® 700 (filled histogram) or rat IgG2a, κ Alexa Fluor® 700 isotype control (open histogram).	https://www.biolegend.com/nl-nl/products/alexa-fluor-700-anti-mouse-cd107a-lamp-1-antibody-16558?GroupID=BLG4966
anti-mouse/human CD11b	PE	101207	BioLegend	M1/70	1 in 200	C57BL/6 mouse bone marrow cells were stained with CD11b (clone M1/70) PE (filled histogram) or rat IgG2b, κ PE isotype control (open histogram) (gated on total cells). Citation: Kisielow J, et al. 2019. Nat Immunol. 1.286111111.	https://www.biolegend.com/de-at/products/pe-anti-mouse-human-cd11b-antibody-349?GroupID=BLG10552
anti-mouse CD25	PE	102007	BioLegend	PC61	1 in 200	Con A-stimulated (3 days) BALB/c mouse splenocytes stained with PC61 PE. Citation: Baptista AP et al. 2019. Immunity. 50(5):1188-1201	https://www.biolegend.com/fr-fr/products/pe-anti-mouse-cd25-antibody-424
anti-mouse CD28		BE0015-1	Bio X Cell	37.51	1ug/ml	Citation: Ron-Harel, N., et al. 2016. Cell Metab 24(1): 104-117.	https://bioxcell.com/invivomab-anti-mouse-cd28-be0015-1?gad_source=1&gclid=CjwKCAjwrlixBhBbEiwACEqDJSXgTugJdpBxoZiQLORHTNmPqh86E40bY0INkAtCpBQizjv5SMPlWhoCS38QAvD_BwE#tab_references
anti-mouse CD3ε		BE0001-1	Bio X Cell	145-2C11 f(ab')2 Fragments	5ug/ml	Citation: Shiheido, H., et al. 2014. Eur J Immunol 44(6): 1770-1780	https://bioxcell.com/invivomab-anti-mouse-cd3e-f-ab-2-fragment-be0001-1fab#tab_specifications
anti-mouse CD4	Alexa Fluor 700	100429	BioLegend	GK1.5	1 in 200	C57BL/6 mouse splenocytes stained with CD4 (clone GK1.5) Alexa Fluor® 700 (filled histogram) or rat IgG2b, κ Alexa Fluor® 700 isotype control (open histogram). Citation: Qi Z, et al. 2022. Nat Commun. 13:182.	https://www.biolegend.com/fr-lu/products/alexa-fluor-700-anti-mouse-cd4-antibody-3385?GroupID=BLG4745
anti-mouse CD4	BUV737	612844	BD Biosciences	RM4.5	1 in 300	Two-color flow cytometric analysis of CD4 expression on mouse splenocytes. Mouse splenic leucocytes were preincubated with Purified Rat Anti-Mouse CD16/CD32 antibody (Mouse BD Fc Block™) (Cat. No. 553141/553142). The cells were then stained with APC Hamster Anti-Mouse CD3e antibody (Cat. No. 553066/561826) and either BD Horizon™ BUV737 Rat IgG2a, κ Isotype Control (Cat. No. 612760; Left Plot) or BD Horizon BUV737 Rat Anti-Mouse CD4 antibody (Cat. No. 612843/612844; Right Plot) at 0.5 µg/test. BD Via-Probe™ Cell Viability 7-AAD Solution (Cat. No. 555815/555816) was added to cells right before analysis. The two-color contour plot showing the correlated expression of CD4 (or Ig Isotype control staining) versus CD3e was derived from gated events with the forward and side light-scatter characteristics of viable (7-AAD-negative) splenic leucocytes. Flow cytometry and data analysis were performed using a BD LSRFortessa™ Cell Analyzer System and FlowJo™ software. Data shown on this Technical Data Sheet are not lot specific.	https://www.bdbiosciences.com/en-us/products/reagents/flow-cytometry-reagents/research-reagents/single-color-antibodies-ruo/buv737-rat-anti-mouse-cd4.612844
anti-mouse CD44	BV510	103043	BioLegend	IM7	1 in 200	C57BL/6 mouse splenocytes were stained with CD44 (clone IM7) Brilliant Violet 510™. Citation: Harsha Krovi S, et al. 2020. Nat Commun. 4.790277778.	https://www.biolegend.com/en-gb/products/brilliant-violet-510-anti-mouse-human-cd44-antibody-7994?GroupID=BLG4687

Supplementary Table I

anti-mouse CD44	BV650	103049	BioLegend	IM7	1 in 200	C57BL/6 mouse splenocytes were stained with CD44 (clone IM7) Brilliant Violet 650™ (filled histogram) or rat IgG2b, κ Brilliant Violet 650™ isotype control (open histogram). Citation: Hu W, et al. 2021. Nat Immunol. 22:1163.	https://www.biolegend.com/fr-fr/search-results/brilliant-violet-650-anti-mouse-human-cd44-antibody-8923
anti-mouse CD45	BV785	103149	BioLegend	30-F11	1 in 200	C57BL/6 mouse splenocytes were stained with CD45 (clone 30-F11) Brilliant Violet 785™ (filled histogram) or rat IgG2b, κ Brilliant Violet 785™ isotype control (open histogram). Citation: Rogic A, et al. 2021. Nat Commun. 12:6889.	https://www.biolegend.com/nl-be/products/brilliant-violet-785-anti-mouse-cd45-antibody-10636
anti-mouse CD62L	Pacific Blue	104423	BioLegend	MEL-14	1 in 200	C57BL/6 mouse splenocytes were stained with CD3 Alexa Fluor® 488 and CD62L (clone MEL-14) Pacific Blue™ (top) or rat IgG2a Pacific Blue™ isotype control (bottom). Miyauchi E, et al. 2020. Nature. 585:102.	https://www.biolegend.com/en-ie/products/pacific-blue-anti-mouse-cd62l-antibody-3117
anti-mouse CD62L	FITC	161211	BioLegend	W18021D	1 in 200	C57BL/6 mouse splenocytes were stained with anti-mouse CD3ε APC and anti-mouse CD62L (clone W18021D) FITC (left) or rat IgG2a, κ FITC isotype control (right).	https://www.biolegend.com/fr-ch/products/fitc-anti-mouse-cd62l-antibody-23532
anti-mouse CD69	Alexa Fluor 647	104517	BioLegend	H1.2F3	1 in 200	PMA+ionomycin-stimulated (6 hours) C57BL/6 mouse splenocytes stained with H1.2F3 Alexa Fluor® 647. Citation: enechet AP, et al. 2019. Nature. 574:200.	https://www.biolegend.com/ja-jp/products/alexa-fluor-647-anti-mouse-cd69-antibody-3184
anti-mouse CD69	PerCP	104520	BioLegend	H1.2F3	1 in 50	PMA + ionomycin-stimulated (6 hrs) C57BL/6 splenocytes stained with H1.2F3 PerCP. Citation: Medler TR et al. 2018. Cancer cell. 34(4):561-578.	https://www.biolegend.com/en-ie/products/percp-anti-mouse-cd69-antibody-4401
anti-mouse CD69	PE	104508	BioLegend	H1.2F3	1 in 200	PMA+ionomycin-stimulated C57BL/6 mouse splenocytes (6 hours) stained with H1.2F3 PE. Glasner A,et al. 2017. Sci Rep.. 10.1038/s41598-017-12998-w.	https://www.biolegend.com/nl-nl/products/pe-anti-mouse-cd69-antibody-265
anti-mouse CD8a	Pacific Blue	100725	BioLegend	53-6.7	1 in 200	C57BL/6 mouse splenocytes were stained with CD8 (clone 53-6.7) Pacific Blue™ (filled histogram) or rat IgG2a, κ Pacific Blue™ isotype control (open histogram). Citation: Logan K Smith et al. 2018. Immunity. 48(2):299-312.	https://www.biolegend.com/de-at/products/pacific-blue-anti-mouse-cd8a-antibody-2856
anti-mouse CD8a	BUV395	565968	BD Biosciences	53-6.7	1 in 300	Two-color flow cytometric analysis of CD8a expression on mouse splenocytes. Mouse splenic leucocytes were preincubated with Purified Rat Anti-Mouse CD16/CD32 antibody (Mouse BD Fc Block™) (Cat. No. 553141/553142). The cells were then stained with APC Hamster Anti-Mouse CD3e (Cat. No. 553066/561826) and BD Horizon™ BUV395 Rat Anti-Mouse CD8a (Cat. No. 563786/565968) antibodies. The two-color fluorescence dot plot shows the correlated expression patterns of CD8a versus CD3e for gated events with the forward and side light-scatter characteristic of viable splenic leucocytes. Flow cytometric analysis was performed using a BD™ LSR II Flow Cytometer System. *18 publications*	https://www.bdbiosciences.com/en-us/products/reagents/flow-cytometry-reagents/research-reagents/single-color-antibodies-ruo/buv395-rat-anti-mouse-cd8a.565968
anti-mouse CD8a	FITC	100705	BioLegend	53-6.7	1 in 200	C57BL/6 mouse splenocytes were stained with CD8 (clone 53-6.7) FITC (filled histogram) or rat IgG2a, κ FITC isotype control (open histogram). Citation: Radtke AJ, et al. 2020. Proc Natl Acad Sci U S A. 117:33455-65. (SB)	https://www.biolegend.com/de-de/products/fitc-anti-mouse-cd8a-antibody-153
anti-mouse CD8a	PE	100708	BioLegend	53-6.7	1 in 200	C57BL/6 mouse splenocytes were stained with CD8 (clone 53-6.7) PE (filled histogram) or rat IgG2a, κ PE isotype control (open histogram). Citation: Li Z, et al. 2022. Nat Commun. 13:1845.	https://www.biolegend.com/fr-ch/products/pe-anti-mouse-cd8a-antibody-155?GroupID=BLG2559
anti-mouse CD8a		98941	Cell Signaling Technology	D4W2Z	1 in 1000	Western blot analysis of extracts from mouse CD8+ T cells, mouse spleen, EL4 cells, and Raw264.7 cells using CD8α (D4W2Z) XP® Rabbit mAb (upper), and β-Actin (D6A8) Rabbit mAb #8457 (lower). The correct band is shown.	https://www.cellsignal.com/products/primary-antibodies/cd8a-d4w2z-xp-rabbit-mab/98941
anti-mouse Fas	BV605	152612	BioLegend	SA367H8	1 in 200	C57BL/6 mouse splenocytes were stained with CD3 APC and with CD95 (SA367H8) Brilliant Violet 605™ (left) or Mouse IgG1, κ Brilliant Violet 605™ isotype control (right). Citation: Yang Y, et al. 2021. Nat Commun. 12:525.	https://www.biolegend.com/de-de/products/brilliant-violet-605-anti-mouse-cd95-fas-antibody-15412
anti-mouse Foxo1	Alexa Fluor 488	58223S	Cell Signaling Technology	C29H4	1 ul per sample	Flow cytometric analysis of Jurkat cells (blue) and IGROV-1 cells (green) using FoxO1 (C29H4) Rabbit mAb (Alexa Fluor® 488 Conjugate) (solid lines) or concentration-matched Rabbit (DA1E) mAb IgG XP® Isotype Control (Alexa Fluor® 488 Conjugate) #2975 (dashed lines). citation: Baessler, et. al. 2022. Sci Adv	https://www.cellsignal.com/products/antibody-conjugates/foxo1-c29h4-rabbit-mab-alexa-fluor-488-conjugate/58223
anti-mouse IFNγ	biotin	505804	BioLegend	XMG1.2	2ug/ml	Citation: Xu G, et al. 2007. J. Immunol. 179:5358.	https://www.biolegend.com/nl-nl/products/biotin-anti-mouse-ifn-gamma-antibody-994

Supplementary Table I

anti-mouse IFNγ		505702	BioLegend	R4-6A2	2ug/ml	Citation: Xu G, et al. 2007. J. Immunol. 179:5358.	https://www.biolegend.com/nl-be/products/purified-anti-mouse-ifn-gamma-antibody-987
anti-mouse MCJ					1 in 1000	the antibody is verfied in previously study: Hatle et al. 2013. Mol Cell Biol. 33(11): 2302–2314.	
anti-mouse PD-1	PE-Cy7	109109	BioLegend	RMP1-30	1 in 200	Con A-stimulated (day-3) Balb/c mouse splenocytes stained with RMP1-30 PE/Cyanine7. Citation: Fu G, et al. 2021. Nature. 595:724.	https://www.biolegend.com/fr-ch/products/pe-cyanine7-anti-mouse-cd279-pd-1-antibody-3612
anti-mouse PD-1	Alexa Fluor 647	109117	BioLegend	RMP1-30	1 in 200	Con A (three-days) activated C57BL/6 splenocytes were stained with CD3 PE and CD279 (clone RMP1-30) Alexa Fluor® 647 (top) or rat IgG2b, κ Alexa Fluor® 647 isotype control (bottom). Citation: Turner JA, et al. 2020. Immunity. 53:1202.	https://www.biolegend.com/de-at/products/alexa-fluor-647-anti-mouse-cd279-pd-1-antibody-12480
anti-mouse T-bet	BV605	644817	BioLegend	4B10	2ul per sample	Human peripheral blood lymphocytes were surface stained with CD3 APC and then treated with True-Nuclear™ Transcription Factor Buffer Set (Cat# 424401). Cells were then stained with T-bet (clone 4B10) Brilliant Violet 605™ (top) or mouse IgG1, κ Brilliant Violet 605™ isotype control (bottom). Citation: Witkowski M, et al. 2021. Nature. 600:295.	https://www.biolegend.com/fr-ch/products/brilliant-violet-605-anti-t-bet-antibody-7907?GroupID=BLG6433
anti-mouse Tcf1	Alexa Fluor 594	35972S	Cell Signaling Technology	C63D9	1ul per sample	Flow cytometric analysis of U-937 cells (blue) and Jurkat cells (green) using TCF1/TCF7 (C63D9) Rabbit mAb (Alexa Fluor® 594 Conjugate) (solid lines) or a concentration-matched Rabbit (DA1E) mAb IgG XP® Isotype Control (Alexa Fluor® 594 Conjugate) #8760 (dashed lines).	https://www.cellsignal.com/products/antibody-conjugates/tcf1-tcf7-c63d9-rabbit-mab-alexa-fluor-594-conjugate/35972
anti-mouse Tim3	BV711	119727	BioLegend	RMT3-23	1 in 200	C57BL/6 mouse splenocytes were stained with anti-mouse CD3ε (clone 145-2C11) PerCP/Fire™ 780 and anti-mouse CD366 (Tim-3) (clone RMT3-23) Brilliant Violet 711™ (left) or rat IgG2a, κ Brilliant Violet 711™ isotype control (right). Citation: Ma S, et al. 2022. Nat Commun. 13:4118.	https://www.biolegend.com/en-gb/products/brilliant-violet-711-anti-mouse-cd366-tim-3-antibody-14918?GroupID=BLG10656
anti-mouse TOX	eFluor 660	50-6502-80	Invitrogen	TXRX10	1.5ul per sample	C57Bl/6 thymocytes were stained with Anti-Mouse CD4 eFluor® 450 (Product # 48-0042-82) and Anti-Mouse CD8a FITC (Product # 11-0081-82), followed by intracellular staining with 0.5 µg of Anti-Human/Mouse TOX eFluor® 660 using the Foxp3/Transcription Factor Buffer Set (Product # 00-5523-00) and protocol. The histogram (right) demonstrates staining of TOX on CD4+CD8+ (blue histogram), CD4loCD8lo (green histogram), and CD4+CD8lo (purple histogram) thymocytes. Cell populations were gated as indicated on the dot plot (left). Cells in the lymphocyte gate were used for analysis.	https://www.thermofisher.com/antibody/product/TOX-Antibody-clone-TXRX10-Monoclonal/50-6502-82
anti-mouse TCR Vα2	FITC	553288	BD Bioscience	B20.1	1 in 200	Flow cytometric analysis of Vα2 TCR expression on mice peripheral lymphocytes. BALB/c lymph node cells were incubated simultaneously with PE Rat Anti-Mouse CD8b.2 (Cat. No. 553041), PE Rat Anti-Mouse CD4 (Cat. No. 553730/561829/557308) and FITC Rat Anti-Mouse Vα2 TCR (Cat. No. 553288/562085). The contour plot depicting CD4 versus Vα2 TCR expression was derived from gated events with the side and forward light-scatter characteristics of viable lymphocytes. Flow cytometry was performed on a FACScan™ system.	https://www.bdbiosciences.com/en-us/products/reagents/flow-cytometry-reagents/research-reagents/single-color-antibodies-ruo/fitc-rat-anti-mouse-v-2-tcr.553288
