# Supplementary material for: Deleting the mitochondrial respiration negative regulator MCJ enhances the efficacy of CD8+ T cell adoptive therapies in pre-clinical studies
Source: Nat Commun. 2024 May 24;15:4444. doi: 10.1038/s41467-024-48653-y (PMC11126743; doi:10.1038/s41467-024-48653-y)
Supplement: Supplementary file 3 — Reporting Summary [file 41467_2024_48653_MOESM3_ESM.pdf]

nature portfolio

Corresponding author(s): *Double-anonymous peer review submissions: write DAPR and your manuscript number here instead of author names.*

Last updated by author(s): *YYYY-MM-DD*

# Reporting Summary

Nature Portfolio wishes to improve the reproducibility of the work that we publish. This form provides structure for consistency and transparency in reporting. For further information on Nature Portfolio policies, see our Editorial Policies and the Editorial Policy Checklist.

## Statistics

For all statistical analyses, confirm that the following items are present in the figure legend, table legend, main text, or Methods section.

| n/a | Confirmed | |
|---|---|---|
| ☐ | ☒ | The exact sample size (*n*) for each experimental group/condition, given as a discrete number and unit of measurement |
| ☐ | ☒ | A statement on whether measurements were taken from distinct samples or whether the same sample was measured repeatedly |
| ☐ | ☒ | The statistical test(s) used AND whether they are one- or two-sided<br>*Only common tests should be described solely by name; describe more complex techniques in the Methods section.* |
| ☐ | ☒ | A description of all covariates tested |
| ☐ | ☒ | A description of any assumptions or corrections, such as tests of normality and adjustment for multiple comparisons |
| ☐ | ☒ | A full description of the statistical parameters including central tendency (e.g. means) or other basic estimates (e.g. regression coefficient) AND variation (e.g. standard deviation) or associated estimates of uncertainty (e.g. confidence intervals) |
| ☐ | ☒ | For null hypothesis testing, the test statistic (e.g. *F*, *t*, *r*) with confidence intervals, effect sizes, degrees of freedom and *P* value noted<br>*Give P values as exact values whenever suitable.* |
| ☒ | ☐ | For Bayesian analysis, information on the choice of priors and Markov chain Monte Carlo settings |
| ☒ | ☐ | For hierarchical and complex designs, identification of the appropriate level for tests and full reporting of outcomes |
| ☐ | ☒ | Estimates of effect sizes (e.g. Cohen's *d*, Pearson's *r*), indicating how they were calculated |

*Our web collection on statistics for biologists contains articles on many of the points above.*

## Software and code

Policy information about availability of computer code

| | |
|---|---|
| Data collection | The DICE (Database of Immune Cell Expression, Expression quantitative trait loci (eQTLs) and Epigenomics) were used to analyze the data in this study |
| Data analysis | Graphpad Prism, versions 8 and 10 and R were used to analyze data. |

For manuscripts utilizing custom algorithms or software that are central to the research but not yet described in published literature, software must be made available to editors and reviewers. We strongly encourage code deposition in a community repository (e.g. GitHub). See the Nature Portfolio guidelines for submitting code & software for further information.

## Data

Policy information about availability of data

All manuscripts must include a data availability statement. This statement should provide the following information, where applicable:

- Accession codes, unique identifiers, or web links for publicly available datasets
- A description of any restrictions on data availability
- For clinical datasets or third party data, please ensure that the statement adheres to our policy

The data generated in this study are available within the article and its supplementary data files.
The human MCJ expression data analyzed in this study were obtained from the published database, DICE (database of immune cell expression, expression quantitative trait loci [eQTLs], and epigenomics). The bulk RNA-seq data is available in the GEO database. There is no new code generated in this study.

## Research involving human participants, their data, or biological material

Policy information about studies with human participants or human data. See also policy information about sex, gender (identity/presentation), and sexual orientation and race, ethnicity and racism.

| | |
|---|---|
| Reporting on sex and gender | *Use the terms sex (biological attribute) and gender (shaped by social and cultural circumstances) carefully in order to avoid confusing both terms. Indicate if findings apply to only one sex or gender; describe whether sex and gender were considered in study design; whether sex and/or gender was determined based on self-reporting or assigned and methods used. Provide in the source data disaggregated sex and gender data, where this information has been collected, and if consent has been obtained for sharing of individual-level data; provide overall numbers in this Reporting Summary. Please state if this information has not been collected. Report sex- and gender-based analyses where performed, justify reasons for lack of sex- and gender-based analysis.* |
| Reporting on race, ethnicity, or other socially relevant groupings | *Please specify the socially constructed or socially relevant categorization variable(s) used in your manuscript and explain why they were used. Please note that such variables should not be used as proxies for other socially constructed/relevant variables (for example, race or ethnicity should not be used as a proxy for socioeconomic status). Provide clear definitions of the relevant terms used, how they were provided (by the participants/respondents, the researchers, or third parties), and the method(s) used to classify people into the different categories (e.g. self-report, census or administrative data, social media data, etc.) Please provide details about how you controlled for confounding variables in your analyses.* |
| Population characteristics | *Describe the covariate-relevant population characteristics of the human research participants (e.g. age, genotypic information, past and current diagnosis and treatment categories). If you filled out the behavioural & social sciences study design questions and have nothing to add here, write "See above."* |
| Recruitment | *Describe how participants were recruited. Outline any potential self-selection bias or other biases that may be present and how these are likely to impact results.* |
| Ethics oversight | *Identify the organization(s) that approved the study protocol.* |

Note that full information on the approval of the study protocol must also be provided in the manuscript.

# Field-specific reporting

Please select the one below that is the best fit for your research. If you are not sure, read the appropriate sections before making your selection.

[x] Life sciences  [ ] Behavioural & social sciences  [ ] Ecological, evolutionary & environmental sciences

For a reference copy of the document with all sections, see nature.com/documents/nr-reporting-summary-flat.pdf

# Life sciences study design

All studies must disclose on these points even when the disclosure is negative.

| | |
|---|---|
| Sample size | Pilot and published studies were used to determine the sample size. The mean and variance between groups was sufficient to determine if differences between groups were significant. |
| Data exclusions | The data considered as outlier based on statistically calculation were excluded. Within the Seahorse analyses, some exclusions were performed due to technical difference due to instrument (for instance, the known edge effect in plates or wells that may not get injected properly). |
| Replication | Multiple experiments were performed to verify the reproducibility of the experimental finding. All attempts at reproducibility were successful. |
| Randomization | The mice were allocated to experimental groups randomly. The in vivo experiments were done in mismatching condition if possible. Various ages and both sexes of the mice were used in the in vivo studies. |
| Blinding | Due to our randomization process, each animal cage contained both control and treated mice in an unpredictable order. |

# Reporting for specific materials, systems and methods

We require information from authors about some types of materials, experimental systems and methods used in many studies. Here, indicate whether each material, system or method listed is relevant to your study. If you are not sure if a list item applies to your research, read the appropriate section before selecting a response.

## Materials & experimental systems

| n/a | Involved in the study | |
|---|---|---|
| ☐ | ☒ | Antibodies |
| ☐ | ☒ | Eukaryotic cell lines |
| ☒ | ☐ | Palaeontology and archaeology |
| ☐ | ☒ | Animals and other organisms |
| ☒ | ☐ | Clinical data |
| ☒ | ☐ | Dual use research of concern |
| ☒ | ☐ | Plants |

## Methods

| n/a | Involved in the study | |
|---|---|---|
| ☒ | ☐ | ChIP-seq |
| ☐ | ☒ | Flow cytometry |
| ☒ | ☐ | MRI-based neuroimaging |

# Antibodies

Antibodies used

anti-hEGFR-PE antibody (BioLegend, Cat#352904, RRID: AB_10896794); anti-mouse CD8 (Cell Signaling Technology, Cat#98941); anti-mouse IFNg capture and biotinylated Abs (BioLegend); anti-G4S staining antibody (Cell Signaling Technology, Cat#38907S); anti-mouse CD8 (BioLegend, Cat#100725, RRID: AB_493425); anti-mouse CD4 (BioLegend, Cat#100429, RRID: AB_493698); anti-mouse CD11b (BioLegend, Cat#101207, RRID: AB_312790); anti-mouse B220 (BioLegend, Cat#103205, RRID: AB_312990); anti-mouse CD44 (BioLegend, Cat#103049, RRID: AB_2562600); anti-mouse CD25 (BioLegend, Cat#102007, RRID: AB_312856); anti-mouse CD69 (BioLegend, Cat#104517, RRID: AB_492848); anti-mouse CD62L (BioLegend, Cat#104423, RRID: AB_493381); anti-mouse PD-1 (BioLegend, Cat#109117, RRID: AB_2566549); anti-mouse Fas antibody (BioLegend, Cat#152612, RRID: AB_2728202); anti-mouse CD8 (BD Biosciences, Cat#565968, RRID: AB_2732919), anti-mouse CD4 (BD Biosciences, Cat#612844), anti-mouse CD45 (BioLegend, Cat#103149, RRID: AB_2564590) and anti-mouseTCR Va2+ (BD Bioscience, Cat#553288, RRID: AB_394759); anti-mouse Tim3 (BioLegend, Cat#119727, RRID: AB_2716208); anti-mouse T-bet (BioLegend, Cat#644817, RRID: AB_11219388), anti-mouse Tcf1 (Cell Signaling Technology, Cat#35972S), anti-mouse Foxo1 (Cell signaling Technology, Cat#58223S), and anti-mouse TOX (Invitrogen, Cat#50-6502-80, RRID: AB_2574265); anti-human CD8 (BioLegend, Cat#300928, RRID: AB_10612929); anti-human CD4 (BioLegend, Cat# 317422, RRID: AB_571941); anti-human CD45RA (BioLegend, Cat# 304120, RRID: AB_493763); anti-human CD45RO (BioLegend, Cat# 304204, RRID: AB_314420); CCR7 (BioLegend, Cat# 353204, AB_10913813); anti-human CD8a (BioLegend, Cat#301046, clone RPA-T8); anti-human CD62L (BD Biosciences. Cat# 741843, clone DREG-56); anti-human CD45RA (BD Biosciences. Cat#550855, clone HI100); anti-human MCJ (generated in lab (Hatle et al, Mol Cell Biol 2007)); anti-GAPDH (Cell signaling); anti-b-actin (Cell signaling); anti-mouse MCJ (generated in lab (Hatle et al., 2007))

Validation

The anti-MCJ, anti-GAPDH, anti-b-actin antibodies gave a band with expected size and were validated by the manufacturers. The antibodies used for flow cytometry were validated with positive and negative control cells and by the manufacturers and were used in other published studies.

# Eukaryotic cell lines

Policy information about cell lines and Sex and Gender in Research

Cell line source(s)

The murine B16-OVA melanoma cell line were generated from Dutton, R. W. group.
The murine E2a pre-B ALL cell line (derived from E2a:PBX1 transgenic mice in the C57Bl/6 background) was provided by Janetta Bijl (Université de Montréal, Montreal, Canada). The E2a cell lines were transduced with lentivirus encoding GFP, and a single cell clone was established by dilutional cloning to generate the E2a GFP+ cell line by Fry, T. J. group.
The human Nalm6 pre-B ALL cell line were from Fry, T. J group.

Authentication

E2a and Nalm6 cell lines were authenticated at NCI
B16-OVA were authenticated by the response of CD8 cells to their OVA expression

Mycoplasma contamination

All cell lines were tested negative for mycoplasma

Commonly misidentified lines
(See ICLAC register)

No cell lines were misidentified

# Animals and other research organisms

Policy information about studies involving animals; ARRIVE guidelines recommended for reporting animal research, and Sex and Gender in Research

Laboratory animals

WT C57BL/6J mice (RRID:IMSR_JAX:000664), OT-I transgenic mice (RRID:IMSR_JAX:003831) and CD4-Cre mice (RRID:IMSR_JAX:022071) were purchased from Jackson Laboratories. MCJ KO mice were previously described46. MCJf/f mice were generated by CRISPR/Cas as described (Henao-Mejia, J. et al., Cold Spring Harb Protoc 2016) using sgRNAs and Cas9 in C57Bl/6 single-cell embryos to insert loxP sites flanking the exon I of mouse MCJ/DnaJC15. One line of MCJf/+ mice was identified, loxP sites insertion was verified and the mice were backcrossed with C57Bl/6 mice first and intercrossed to generate homozygous MCJf/f mice. These mice were then crossed with CD4-Cre transgenic mice. Mice between 3-6 months were used in the study.

Wild animals

No wild animals were used in the study

Reporting on sex

Both sexes were used in the study. No clear differences were observed between 2 sexes in experimental finding

| | |
|---|---|
| Field-collected samples | No field-collected samples were used in the study |
| Ethics oversight | Animal studies were approved by the Institutional Animal Care and Use Committee (IACUC) of the University of Vermont and University of Colorado, and performed following the guidelines. |

Note that full information on the approval of the study protocol must also be provided in the manuscript.

# Plants

| | |
|---|---|
| Seed stocks | *Report on the source of all seed stocks or other plant material used. If applicable, state the seed stock centre and catalogue number. If plant specimens were collected from the field, describe the collection location, date and sampling procedures.* |
| Novel plant genotypes | *Describe the methods by which all novel plant genotypes were produced. This includes those generated by transgenic approaches, gene editing, chemical/radiation-based mutagenesis and hybridization. For transgenic lines, describe the transformation method, the number of independent lines analyzed and the generation upon which experiments were performed. For gene-edited lines, describe the editor used, the endogenous sequence targeted for editing, the targeting guide RNA sequence (if applicable) and how the editor was applied.* |
| Authentication | *Describe any authentication procedures for each seed stock used or novel genotype generated. Describe any experiments used to assess the effect of a mutation and, where applicable, how potential secondary effects (e.g. second site T-DNA insertions, mosiacism, off-target gene editing) were examined.* |

# Flow Cytometry

## Plots

Confirm that:

☒ The axis labels state the marker and fluorochrome used (e.g. CD4-FITC).

☒ The axis scales are clearly visible. Include numbers along axes only for bottom left plot of group (a 'group' is an analysis of identical markers).

☒ All plots are contour plots with outliers or pseudocolor plots.

☒ A numerical value for number of cells or percentage (with statistics) is provided.

## Methodology

| | |
|---|---|
| Sample preparation | For mitochondrial membrane potential (MMP) analysis the cells were stained with TMRE for 20min at 37dC. For mitochondrial mass analysis, the CD8 CAR-T cells were stained with Mitotracker 633 (Thermo Fisher Scientific) for 30 minutes at 37dC. For reactive oxygen species (ROS) analysis, the assay was performed by staining cells with MitoSOX Red (Thermo Fisher Scientific, Cat#M36008) for 10 minutes at 37dC. The anti-hEGFR (BioLegend, Cat#352904, RRID: AB_10896794) or anti-G4S staining antibody (Cell Signaling Technology, Cat#38907S) was added at the last 5-10 min of the incubation with the corresponding dye to identify the CAR positive population. The cells were washed twice and examined on the flow cytometer.<br><br>To determine the surface markers the cells were stained at 4dC for 30min, and washed with FACS buffer twice before running on the flow cytometer. We followed the recommended procedure from manufacturers for intracellular staining (BD PharmingenTM Transcription factor buffer set, Cat#562574). |
| Instrument | Northern Lights and Aurora (Cytek Biosciences). LSRFortessa (BD Biosciences). |
| Software | FlowJo |
| Cell population abundance | The post-sort populations were verified by the flow cytometry for the purity |
| Gating strategy | The live population of cells were gating based on FSC and SSC or based on the viability staining. The positive and negative gates were draw based on the distinct population within the samples, as well as based on the FMO or the unstained cells. |

☒ Tick this box to confirm that a figure exemplifying the gating strategy is provided in the Supplementary Information.
